# Isolation and characterization of haploid heterothallic beer yeasts

**DOI:** 10.1007/s00253-024-13397-8

**Published:** 2025-01-22

**Authors:** Jennifer Badura, Beatrice Bernardi, Judith Muno-Bender, Katrin Matti, Kerstin Zimmer, Jürgen Wendland

**Affiliations:** 1 Department of Microbiology and Biochemistry, Hochschule Geisenheim University, Von-Lade-Straße 1, 65366 Geisenheim, Germany; 2 Geisenheim Yeast Breeding Center, Hochschule Geisenheim University, Von-Lade-Straße 1, 65366 Geisenheim, Germany; 3 Present Address: Formo Bio GmbH, Weißmüllerstraße 50 60314, Frankfurt Am Main, Germany

**Keywords:** Fermentation, Lager yeast, Domestication, Ploidy, Breeding, *HO* gene

## Abstract

**Abstract:**

Improving ale or lager yeasts by conventional breeding is a non-trivial task. Domestication of lager yeasts, which are hybrids between *Saccharomyces cerevisiae* and *Saccharomyces eubayanus*, has led to evolved strains with severely reduced or abolished sexual reproduction capabilities, due to, e.g. postzygotic barriers. On the other hand, *S. cerevisiae* ale yeasts, particularly Kveik ale yeast strains, were shown to produce abundant viable spores (~ 60%; Dippel et al. Microorganisms 10(10):1922, 2022). This led us to investigate the usefulness of Kveik yeasts for conventional yeast breeding. Surprisingly, we could isolate heterothallic colonies from germinated spores of different Kveik strains. These strains presented stable mating types in confrontation assays with pheromone-sensitive tester strains. Heterothallism was due to inactivating mutations in their *HO* genes. These led to amino acid exchanges in the Ho protein, revealing a known G223D mutation and also a novel G217R mutation, both of which abolished mating type switching. We generated stable *MAT****a*** or *MATα *lines of four different Kveik yeasts, named Odin, Thor, Freya and Vör. Analyses of bud scar positions in these strains revealed both axial and bipolar budding patterns. However, the ability of Freya and Vör to form viable meiotic offspring with haploid tester strains demonstrated that these strains are haploid. Fermentation analyses indicated that all four yeast strains were able to ferment maltose and maltotriose. Odin was found to share not only mutations in the *HO* gene, but also inactivating mutations in the *PAD1* and *FDC1* genes with lager yeasts, which makes this strain POF-, i.e. not able to generate phenolic off-flavours, a key feature of lager yeasts. These haploid ale yeast-derived strains may open novel avenues also for generating novel lager yeast strains by breeding or mutation and selection utilizing the power of yeast genetics, thus lifting a block that domestication of lager yeasts has brought about.

**Key points:**

*• Haploid Kveik ale yeasts with stable MAT* ***a*** *and MATα mating types were isolated.*

*• Heterothallic strains bear mutant HO alleles leading to a novel inactivating G217R amino acid change.*

*• One strain was found to be POF- due to inactivating mutations in the PAD1 and FDC1 gene rendering it negative for phenolic off-flavor production.*

*• These strains are highly accessible for beer yeast improvements by conventional breeding, employing yeast genetics and mutation and selection regimes.*

**Supplementary Information:**

The online version contains supplementary material available at 10.1007/s00253-024-13397-8.

## Introduction

Beer is the most popular fermented beverage worldwide. It is produced in various fashions of which lager beer represents the highest volume followed by ale. Both types of beers are fermented by distinct yeasts. Lager beer is produced at lower temperatures (6–14 °C) and ale at higher temperatures (15–25 °C), while Kveik ale yeasts used in Western Norwegian farmhouse brewing were used at even higher temperatures (25–40 °C; Vidgren et al. 2010; Preiss et al. 2018). Lager beer yeasts are descendants from an ancestral hybridization event between a *Saccharomyces cerevisiae* and a *Saccharomyces eubayanus* yeast strain and thus share a common origin (Walther et al. 2014; Okuno et al. 2016; Gorter de Vries et al. 2019; Salazar et al. 2019). Due to the restricted and continuous utilization of lager yeasts at brewing sites, local selection generated evolutionary trajectories that resulted in the formation of two main lager yeast groups, Saaz and Frohberg (Gibson et al. 2013). Saaz lager yeasts are triploid/aneuploid with a majority of DNA derived from the *S. eubayanus* parent. Frohberg yeasts, on the other hand, are tetraploid/aneuploid strains with a ~ 2:2 distribution of *S. cerevisiae* and *S. eubayanus* genomes (Dunn and Sherlock 2008; Walther et al. 2014; Wendland 2014; Van den Broek et al. 2015).

The quality of beer brewing greatly benefitted from the isolation of pure cultures and the maintenance of these strains in propagation tanks, which was started in 1883 by Emil Christian Hansen’s isolation of Unterhefe no. 1, later known as *Saccharomyces carlsbergensis* (Hansen 1883; Lin et al. 2021). However, the use of pure cultures and the selection of single strains put a strong bottleneck in place against further diversification and evolution of these yeasts (Steensels et al. 2014; Gallone et al. 2016; Hutzler et al. 2023). The hybrid nature of lager yeasts results in postzygotic sterility rendering it extremely challenging to improve lager yeast strains by conventional breeding (Garcia Sanchez et al. 2012).

Diverse strategies to overcome lager yeast hybrid sterility have been explored to improve their fertility, including rare mating and protoplast fusion or genetic engineering (Gunge and Nakatomi 1972; Krogerus et al. 2016, 2017a, 2021; Sipiczki et al. 2020; Naseeb et al. 2021; Molinet et al. 2022; Mozzachiodi et al. 2022). However, strains generated through modern engineering tools lack consumer acceptance and have not been employed in production (Gibson et al. 2017). Due to the very low sporulation and spore germination efficiency, breeding with lager yeast spore-derived clones is very tedious. An alternative approach is to regenerate *S. cerevisiae* × *S. eubayanus* hybrids. The de novo formation of lager yeast hybrids became possible with the isolation of wild *S. eubayanus* strains (Libkind et al. 2011; Hebly et al. 2015; Krogerus et al. 2017a,b; 2021; Turgeon et al. 2021). Using the *S. eubayanus* diversity and *S. cerevisiae* wine yeasts, novel interspecies hybrids were obtained manually by direct mating of germinated spores (Molinet et al. 2024). However, using wild *S. eubayanus* strains may also introduce undesirable phenotypic traits such as phenolic off-flavor production (POF +) or a reduced ability to utilize maltotriose (Turgeon et al. 2021). Thus, the fermentation industry is left without straightforward, high-throughput tools based on molecular yeast breeding for the development of novel yeast strains responding to modern challenges, including climate change and evolving consumer preferences (Knudsen et al. 2022).

**Fig. 1:**
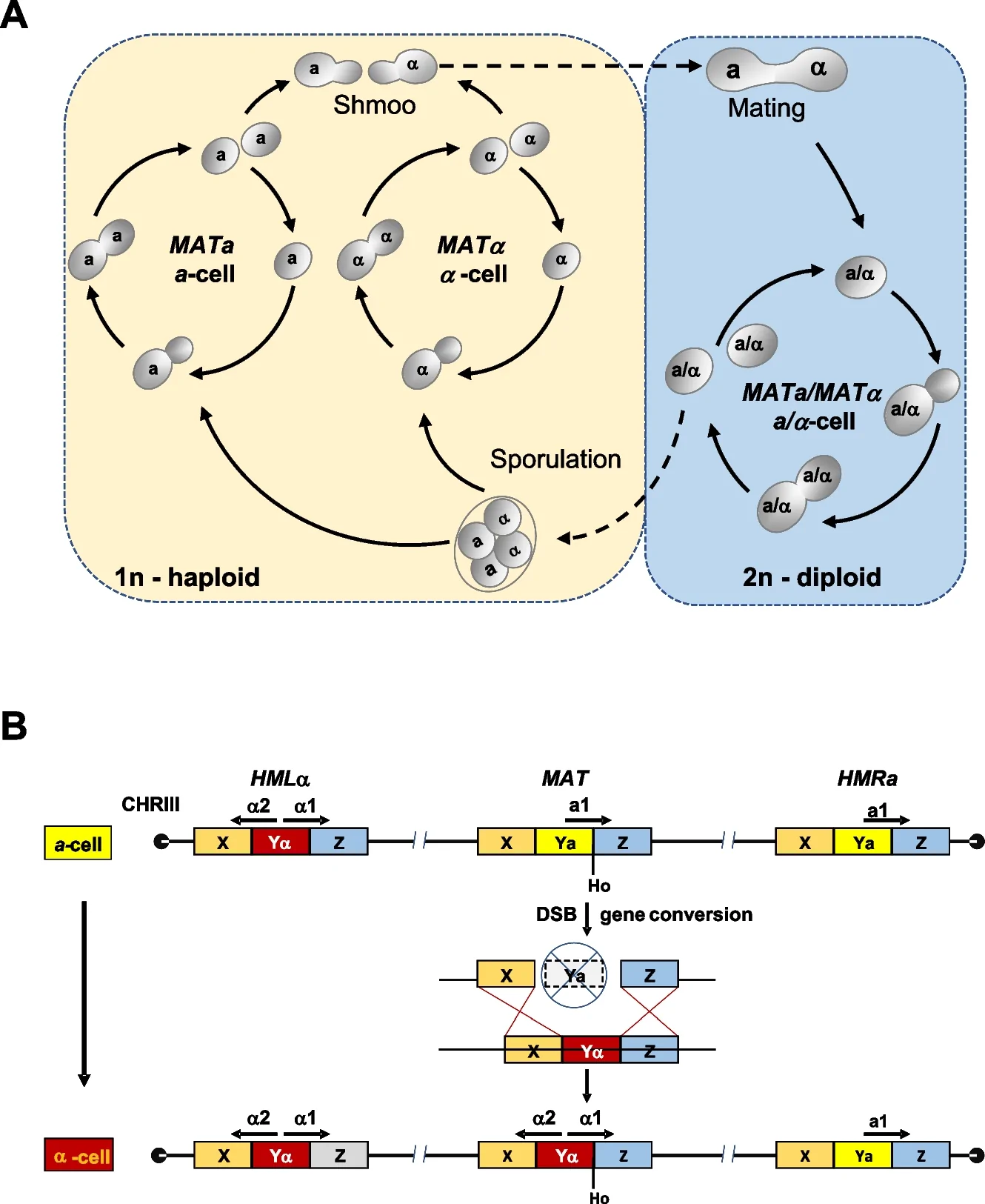
Life cycle of *Saccharomyces cerevisiae*. **A** Haploid **a** and α cells may proliferate by budding or interact via pheromone signalling to mate and form **a**/*α* diploid cells. Diploid cells proliferate or under adverse conditions develop into asci, enter meiotic divisions and form four ascospores per ascus, two with **a** and α mating type each. An **a** cell is determined by *MAT****a*** and an α cell by *MAT*α, while diploids carry both mating type loci and, hence, are *MAT****a****/α*. **B** In haploid mother cells, the Ho endonuclease initiates a mating type switch by introducing a double-strand break (DSB) at the *MAT* locus in the G_1_ phase of the cell cycle. This form of homothallism allows rapid diploidization of a colony derived from a single spore in the wildtype (Haber 2012)

Conventional breeding of beer yeasts relies on the exploitation of the sexual life cycle of *Saccharomyces* (Fig. 1A). Haploid spores carry either *MAT****a*** or *MATα* mating type loci and germinate to give rise to haploid **a** or α cells, respectively. Haploid cells of opposite mating type fuse to generate diploid **a**/α cells. Diploid cells cannot mate but can develop into asci under nutrient-limiting conditions in which meiotic divisions generate four spores (Knop 2006). Haploid cells show an axial budding pattern. This allows budding only at the proximal pole, while diploid cells can use both cell poles for budding and generate the bipolar budding pattern (Freifelder 1960; Chant and Pringle 1995). In order to generate both cell types from a single cell, haploid mother cells initiate a mating type switch. This requires the Ho endonuclease, which is expressed only in mother cells, but not in daughter cells. Ho generates a double-strand break (DSB) at the *MAT* locus and initiates a gene conversion event resulting in the replacement of mating type locus DNA with that of the opposite mating type stored in a silent cassette at the telomere ends of *CHRIII* (Fig. 1B; Russell et al. 1986; Nasmyth and Shore 1987; Lee and Haber 2015). *S. cerevisiae* laboratory yeast strains are generally heterothallic and deficient in mating-type switching due to inactivating mutations in *HO* or due to deletion of the *HO* gene, whereas natural yeast isolates are generally homothallic (Katz Ezov et al. 2010). The laboratory yeast strain S288C bears a specific inactivating *HO* mutation (G667A) leading to an amino acid exchange of glycine at position 223 to serine in the resulting protein. This exchange abolishes enzymatic function. Thus, homothallism adds to the difficulty in obtaining yeast strains with stable mating types for targeted yeast breeding.

Recently, Kveik yeast strains have been described that, even though related to *S. cerevisiae* ale yeasts, belong to a distinct group of beer yeasts, termed ‘European Farmhouse’ ale yeasts (Preiss et al. 2024). They are closely related to, but distinct from ale yeasts grouped into the ‘Beer 1’ group (Gallone et al. 2016; Preiss et al. 2018, 2024). Kveik strains harbor several interesting traits, among them fast fermentation at high temperatures (that is Kveik = quick), good flocculation properties, desiccation resistance/osmotolerance and lack of phenolic off-flavor production (Preiss et al. 2018; Foster et al. 2022; Garshol 2020; Garshol 2022). With these properties, Kveik yeasts would be ideal starter cultures for a range of fermented beverage applications including both lager and ale type of beers. First trials with different beer varieties (stout, Indian Pale Ale) and whisky were already reported (Kawa-Rygielska et al. 2021, 2022; Waymark and Hill 2021). A significant drawback for breeding efforts and genetic alterations, however, is the reported tetraploidy/aneuploidy of Kveik isolates (Preiss et al. 2018, 2024).

Kveik yeast sporulates well and the viability of these spores was found to be exceptionally high amongst beer yeasts (> 60%; Dippel et al. 2022). Therefore, we wanted to explore their breeding potential in more detail. Kveik spores were found to yield stable heterothallic mating-competent strains of both mating types. Spore clone derivates, termed Freya and Vör, presented axial patterns and produced viable meiotic offspring when crossed with haploid tester strains as mating partners. Heterothallism was caused by inactivating mutations in the *HO* gene. One strain, termed Odin, was found to carry inactivating mutations in the *PAD1* and *FDC1* genes indicating a POF- phenotype, i.e. the inability to generate phenolic off-flavor 4-vinyl guaiacol (Mukai et al. 2014). With these novel haploid beer yeast strains the most important shackles of domestication for further strain improvement of traditional beer yeasts have been removed. Odin (*MAT****a***) and Freya (*MATα*) may thus serve as starting strains for new generations of lager and ale starter cultures utilizing the full scope of yeast genetics and allow accessing the biodiversity of *Saccharomyces* yeasts, particularly wine yeasts and their hybrids via conventional yeast breeding.

## Materials and methods

### Strains and growth media

Yeast strains corresponding to *S. cerevisiae* ale yeasts referred to as Kveik and to lager yeasts *Saccharomyces pastorianus* were subcultured in YPD (10 g/L yeast extract, 20 g/L peptone, 20 g/L dextrose) at 25–30 °C. The DH5α bacterial strain (Life Technologies GmbH, Darmstadt, Germany) used for plasmid propagation was grown in 2xYT (16 g/L Bacto peptone, 10 g/L yeast extract, 5 g/L NaCl) at 37 °C. Complete Supplement Mixture (CSM) minimal medium (20 g/L glucose, 1.7 g/L Yeast Nitrogen Base w/o amino acids and without ammonium sulfate, 1.0 g/L asparagine, 0.69 g/L CSM) was used to select for hybrids. For solid media, 20 g/L agar was added before autoclaving. As antibiotics, G418 (200 µg/mL, for the selection of yeast transformants) and ampicillin (100 µg/mL, for the selection of bacterial plasmids) were used. Strains generated and used in this study are hosted in the Geisenheim Yeast Breeding Center (GYBC) collection and are listed in Table 1.

**Table 1:** Strains used in this study

Strain number^1^	Description	Genotype	Source
W34/70	Lager yeast	*Saccharomyces cerevisiae* × *S. eubayanus* hybrid	Weihenstephan, Freising, Germany
W164	Lager yeast	*Saccharomyces cerevisiae* × *S. eubayanus* hybrid	Weihenstephan, Freising, Germany
W195	Lager yeast	*Saccharomyces cerevisiae* × *S. eubayanus* hybrid	Weihenstephan, Freising, Germany
GYBC 145	Kveik 1, Framgarden	Wildtype	Dippel et al. (2022)
GYBC 146	Kveik 2, Strada	Wildtype	Dippel et al. (2022)
GYBC 147	Kveik 3, Tormodgarden	Wildtype	Dippel et al. (2022)
GYBC 148	Kveik 4, Ebbegarden	Wildtype	Dippel et al. (2022)
B003	BY4741	*his3∆1*; *leu2∆0*; *met15∆0*; *ura3∆0*	Euroscarf, Frankfurt, Germany
B006	BY4741	*ade2::kanMX*	Euroscarf, Frankfurt, Germany
B008	BY4742	*his3∆1*; *leu2∆0*; *lys2∆0*; *ura3∆0*	Euroscarf, Frankfurt, Germany
B013	BY4742	*yku70::kanMX*	Euroscarf, Frankfurt, Germany
B025	CEN.PK2.1c	*ura3-52*; *trp1-289*; *leu2-3_113*; *his3∆1*	Euroscarf, Frankfurt, Germany
G213	CEN.PK2.1c	*MAT****a***, *bar1::YES1*	This study
G215	BY4742	*MATα*, *sst2::YES1*	This study
GYBC 706, GYBC 711, GYBC 712, GYBC 713	Spore clones of W164	*MAT****a***	This study
GYBC 714, GYBC 715, GYBC 716, GYBC 717	Spore clones of W195	*MAT****a***	This study
GYBC 315, GYBC 707, GYBC 708, GYBC 709, GYBC 710	Spore clones of W34/70	*MAT****a***	This study
GYBC 244	Odin 2D	*MAT****a***	This study
GYBC 246	Odin 8A (spore clone of GYBC 145)	*MAT****a***	This study
GYBC 249	Odin 17A (spore clone of GYBC 145)	*MAT****a***	This study
GYBC 252	Thor 3B (spore clone of GYBC 146)	*MAT****a***	This study
GYBC 254	Thor 10A (spore clone of GYBC 146)	*MAT****a***	This study
GYBC 256	Thor 15A (spore clone of GYBC 146)	*MAT****a***	This study
GYBC 259	Thor 25D (spore clone of GYBC 146)	*MATα*	This study
GYBC 263	Freya 8C (spore clone of GYBC 147)	*MATα*	This study
GYBC 274	Vör 11D (spore clone of GYBC 148)	*MAT****a***	This study
GYBC 322	Hybrid of GYBC 263 and B006	*MAT****a/****α*	This study
GYBC 323	Hybrid of GYBC 263 and B006	*MAT****a/****α*	This study
GYBC 324	Hybrid of GYBC 274 and B013	*MAT****a/****α*	This study
GYBC 325	Hybrid of GYBC 274 and B013	*MAT****a/****α*	This study
GYBC 355–358	Spore clones of tetrad #2 of GYBC 323	2:2 *MAT****a***:*MATα*	This study
GYBC 359–362	Spore clones of tetrad #1 of GYBC 325	2:2 *MAT****a***:*MATα*	This study
GYBC 363–366	Spore clones of tetrad #2 of GYBC 325	2:2 *MAT****a***:*MATα*	This study
GYBC 367–370	Spore clones of tetrad #2 of GYBC 325	2:2 *MAT****a***:*MATα*	This study

^1^*GYBC* Geisenheim Yeast Breeding Center

### Generation of pheromone-sensitive tester strains

PCR-based gene targeting was used for the deletion of *BAR1* in CEN.PK2.1c (*MAT****a***) and of *SST2* in BY4742 MATα (Jacobs and Lew 2022). The selectable marker gene *YES1* (Kayacan et al. 2019), providing resistance to G418, was amplified from E088 (pGEM-YES1) with primers 776-S1-BAR1 and 777-S2-BAR1 and with primers 780-S1-SST2 and 781-S2-SST2 to add flanking homology regions to the marker gene to achieve targeted gene replacements of *BAR1* and *SST2*, respectively, in *S. cerevisiae*. The lithium acetate/single strand carrier DNA/polyethylene glycol/dimethylsulfoxid (DMSO) method was used for the transformation of *S. cerevisiae* (Schiestl and Gietz 1989). Correct gene deletions were analyzed by diagnostic PCR on the genomic DNA of primary transformants. This generated strains G213 (*MAT****a***, *bar1::YES1*) and G215 (*MATα*, *sst2::YES1*). Primer details are listed in Table 2.

**Table 2:** Primers used in this study

Primer	Sequence (5′ to 3′)*
25-MATalpha	GCACGGAATATGGGACTACTTCG
26-MATa	ACTCCACTTCAAGTAAGAGTTTG
27-MAT	AGTCACATCAAGATCGTTTATGG
776-S1-BAR1-YES1	ATCATACCAAAATAAAAAGAGTGTCTAGAAGGGTCATATAGAAGCTTcgtacgctgcaggtc
777-S2-BAR1-YES1	TTGATATTTATATGCTATAAAGAAATTGTACTCCAGATTTCtctgatatcatcgatgaattcGAG
778-G1-BAR1	GAGATGCGTTGTCCCTG
779-G4-BAR1	CTTGTCGCGTGCCAGATC
780-S1-SST2-YES1	CAATTTGGTAATTAAAGATAGAGTTGTAAGATGGTGGATAGAAGCTTcgtacgctgcaggtc
781-S2-SST2-YES1	GTACCTGAAGATGAGTAAGACTCTCAATGAAATTAGCACTtctgatatcatcgatgaattcGAG
782-G1_SST2	GAACCACCTCCGTTGTCTC
783-G4_SST2	GATTCGTAAGCGCTCACG
#355-G2-YES1	gaatgaatctactggtttgg
#356-G3-YES1	Gtgtcggtatcgcagac
1039-G1-HO	CTATCACCCACTAGTACTACC
1041-F1	GAGCTTTTGAAGGTGAACCTG
1042-I1	CATTGAAGTTAGAGATTTGG
1043-I3	CCATTCTGGAAAGCTGTCAC
1044-I5	GTCATATTGTCGAAGTGGTCAC
1045-I7	GTACCAGAAGCACGTGAAGTG
1046-G4-HO	CTGTTACTGATATGTCTGAGG
1300-PAD1-G1	CATAATGCTGCAAATATAGATTGA
1301-PAD1-G3	TGGCAACCAAGACATACTCTGTTC
1302-PAD1-G4	TTTAGCAAGTAACAAATCAACTCT
1303-PAD1-G6	GAACAGGGCACAACAATCATACCA
1304-FDC1-G1	TTCCTCTGAGTTATTCTATTCTTG
1305-FDC1-G3	CCAATCACTGTTCCTGTGTCATCT
1306-FDC1-G5	TCGGTTCCAGTAGTAAAATGTGAG
1307-FDC1-G7	AAGCATTGAAGACAACGCCTGAAG
1308-FDC1-G4	GAAAGATGGATAGTGTTAATGGCG

*Lowercase letters indicate homology to the *YES1* marker

### Halo assay

In a halo assay, the ability of a query strain to induce a growth arrest in a pheromone-sensitive tester strain is analyzed (Julius et al. 1983). This provides an indication of the mating type of the query strain. As pheromone sensitive tester, strains G213 and G215 were used (see above). These strains were grown overnight and diluted tenfold in H_2_O and 150 µL of each strain was plated on the YPD plate to generate a lawn of cells. After spreading of the tester, strain plates were incubated for 30 min at 30 °C to dry before 2 µL of each of the query strains (~ 1 × 10^6^ cells) was spotted on top. Plates were incubated overnight at 30 °C and then assayed for halo formation. Strains that generated a halo (= zone of growth inhibition) in the tester strains were validated by mating type PCR.

### PCR-amplifications and sequence analyses

Mating-type PCR was done according to Huxley et al. (1990). A universal primer (27-MAT) was paired either with a *MAT****a***-specific primer (26-MATa) generating a PCR product of 544 bp or a *MAT*α-specific primer (25-MATalpha) yielding a product of 404 bp. The *HO* alleles were amplified by PCR with primers 1039-G1-HO and 1046-G4-HO. *PAD1* was amplified with primers 1300-PAD1-G1 and 1302-PAD1-G4 and *FDC1* alleles were amplified with primers 1304-FDC1-G1 and 1308-FDC1-G4, respectively. For sequencing, nested primers were employed. Primers are listed in Table 2. Sequences generated in this study were also deposited with GenBank under accession numbers PQ154469-PQ154480. The S288C genome sequence is available at SGD (http://www.yeastgenome.org/).

### Sporulation and tetrad dissection

Sporulation of yeast strains was carried out with strains pre-grown in YPD for 24 h in baffled flasks at 25 °C under constant rotation (140 rpm). Then, 8 mL of cell suspension of each strain was centrifuged, washed once with sterile H_2_O and resuspended in 1 mL H_2_O, and 100 µL was plated on sporulation plates (2% potassium acetate, 0.22% yeast extract, 0.05% glucose, 0.08% Complete Supplement Mixture (CSM) supplemented with 20 µg/mL adenine and adjusted to pH 7). Plates were incubated at 25 °C for 2–10 days. Dissection of spores was done using a micromanipulator (MSM 400, Singer Instruments, Roadwater, UK) as described (Sherman and Hicks 1991).

To isolate spore clones on a larger scale, cell material was removed from sporulation plates, resuspended in 300 µL H_2_O and treated with zymolyase (1 µg/µL) for 30 min at 30 °C, and then, remaining vegetative cells were inactivated by heat (20 min at 50 °C) and with the addition of 1.2 mL 0.03% Triton X-100 (Sherman 2002). This mixture was incubated overnight at room temperature. Then, aliquots were plated on YPD plates and incubated for 2–3 days at 25 °C.

To determine sporulation efficiency, cells were picked from the sporulation plate and examined under the microscope. For each strain, the sporulation of a total of 400 cells was quantified. The percentage of sporulation efficiency was then calculated. To determine the germination efficiency, 25 tetrads were dissected per strain by micromanipulation. The dissection plates were incubated at 30 °C for 5 days before colonies were counted.

### Microscopy

Microscopic images were acquired using an Axiovert 200 M (Zeiss, Jena, Germany) microscope equipped with an AxioCam MRm camera run by the Axiovision 4 software package. Cells grown overnight in YPD were stained with calcofluor white (10 µg/mL; VWR, Darmstadt, Germany) and imaged using the appropriate filter set.

### Yeast breeding

Sexual crosses of Freya (*MATα*, *LEU2*) with BY4741 (*MAT****a***, *kanMX*, *leu2*) and of Vör (*MAT****a***, *LEU2)* with BY4742, (*MATα*, *kanMX*, *leu2*) were generated. To this end, the strains were propagated separately in YPD; aliquots of strain pairs were mixed and incubated overnight at 28 °C to allow for mating interactions to occur. To select for zygotes, cell mixtures were then plated on a minimal medium lacking leucine (CSM-leu) with the addition of G418 to complement each strain deficiency. Zygotes were validated by mating-type PCR.

### Fermentation setup and analyses

In lab-scale fermentation experiments, yeasts were inoculated at ~ 1 × 10^6^ cells/ml, as determined by counting precultures in a haemocytometer and generating appropriate dilutions thereof, in granulated unhopped malt and fermented at 20 °C using 250 mL tall-tube cylinders covered with aluminium foil. Lab scale fermentations were carried out in triplicate in 150 mL Granmalt wort at 15° Plato stirred with magnetic stirrer bars at 300 rpm (GranMalt AG, Gergenkirchen, Germany). CO_2_ loss was measured daily to monitor fermentation progress as described before (Dippel et al. 2022). At the end of fermentation, samples of 1 mL were pelleted at 13,000 rpm for 10 min by centrifugation, and the supernatant was used to measure residual sugars and ethanol formation via high-performance liquid chromatography (HPLC) as described previously (Dippel et al. 2022; Badura et al. 2023). In short, analyses were performed using an HPLC Agilent Technologies Series 1100 (Agilent Technologies, Steinheim, Germany) provided with an autosampler, a multi-wavelength (MWD) as well as a refractive index detector (RID) and a binary pump. The analytes were separated by using an HPLC column with a length of 250 mm (inside diameter, 4.6 mm; particle size, 5 µm; Allure Organic Acids Column, Restek, Bad Homburg v. d. Höhe, Germany). Sugars and ethanol were detected by the RID. Deionized water with 0.5% ethanol and acidified with 0.0139% concentrated sulphuric acid (95–97%) was used as the isocratic eluent. A column temperature of 46 °C was used at a flow rate of 0.6 mL/min. Analytes were analyzed and integrated and their concentrations were determined via Chemstation software (Agilent, Steinheim, Germany). Samples were prepared by centrifuging at 13,000 rpm for 10 min and then diluting the supernatant fivefold with ultrapure water while adding 55 µL of 10% ethanol.

### Statistical analysis

Fermentations were carried out in triplicate. GraphPad Prism version 10.3.1 (GraphPad Software, San Diego, CA, USA) was used to analyze the data for statistical evaluation via two-tailed unpaired *t*-test with Welch’s correction; a significance of *p* < 0.05 was set to the control (respective parental strain). Agilent software ChemStation for LC systems was used to analyze HPLC data (Agilent, Santa Clara, CA, USA).

## Results

### Isolation and characterization of lager yeast spore clones

Yeast strains with stable mating types, i.e. heterothallic yeast strains that are genotypically either *MAT****a*** or *MATα*, which also exhibit a corresponding phenotypic mating behaviour as **a**-cells or *α*-cells, respectively, are very useful for strain manipulations through conventional yeast breeding. Therefore, we initiated a screen in order to obtain mating competent cell lines from a set of lager yeasts (see Table 1). To this end, we sporulated the strains on sporulation plates and used either microdissection or mass spore isolation to isolate spore clones (= yeast colonies generated by a single spore). Sporulation is a very rare event in lager yeast strains and in our hands occurred at a rate of 0.25% (for W164) to 1.5% (for W195) with asci containing one to three spores, while four-spored asci were not found in these strains. This situation is exacerbated due to the low germination rate of lager yeast spores.

**Fig. 2:**
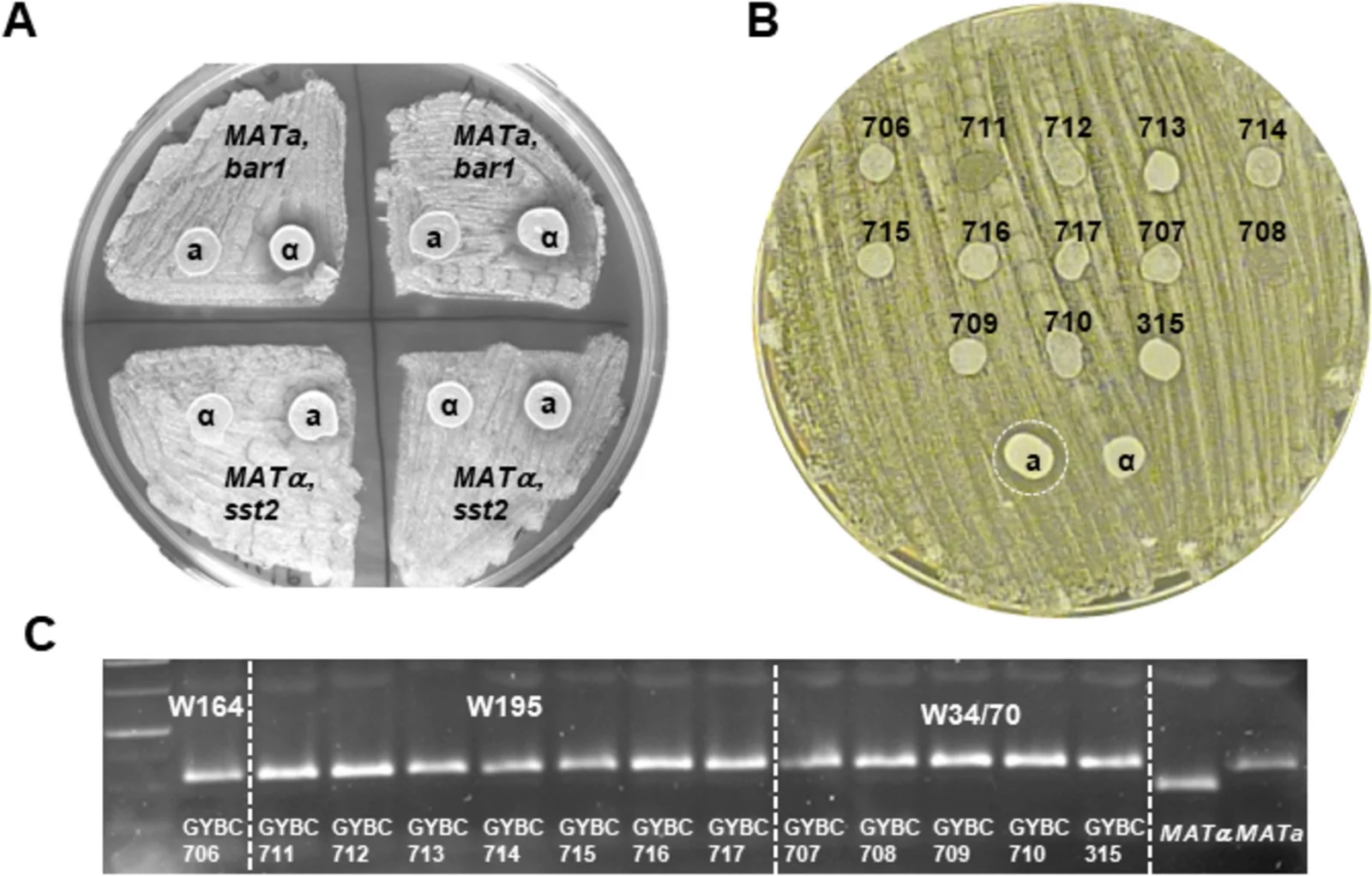
Halo assay for mating type assessment. **A** Use of pheromone-sensitive *MAT****a**** bar1* or *MATα sst2* tester strains in halo assays with query strains. *MATα* query strains will induce larger halos in tester strains due to their more diffusible α-factor. Halos in *MAT****a*** tester strains tend to be very small. **B** A selection of lager yeast spore clones is shown (numbers correspond to GYBC strain collection numbers) that induced halos when used against the *MAT*α tester strain. **C** The mating type configuration of these strains was tested in Mating type PCR/gel electrophoresis and confirmed to be *MAT****a*** in all cases. Spore clones of three lager yeast strains, W164, W195 and W34/70, were tested. *MAT****a*** and *MATα* bands (right lanes) were used as controls; size marker in the left lane

To analyze the mating ability of these spore clones, a halo assay was used (see Materials and Methods). To this end, we generated pheromone-sensitive tester strains by deleting either *BAR1* or *SST2* in the haploid CEN.PK2.1c *MAT****a*** or BY4742 *MATα* strains, respectively. Compatible query strains of opposite mating types will inhibit the growth of cells of the tester strains in close proximity due to the overactivation of the pheromone response signal transduction cascade in these cells. This leads to the formation of a small halo around *MAT****a*** query strains and a bigger halo around *MATα* query strains (Fig. 2A). Using this assay with lager yeast spore clones, we identified strains with stable mating types from the three lager yeast strains W164, W195 and WS34/70. In all cases, the spore clones derived from these strains mated as *MAT****a*** strains (Fig. 2B). Mating type PCR confirmed that these strains harboured only a *MAT****a*** allele at the *MAT* locus. However, for W164 only one spore clone and for W34/70 only five spore clones were isolated; all others were derived from W195 (Fig. 2C). The poor sporulation ability of lager yeasts and our inability to obtain *MATα* spore clones demonstrated the general difficulties in advancing lager yeast strain development through breeding.

### Isolation and characterization of Kveik spore clones

**Fig. 3:**
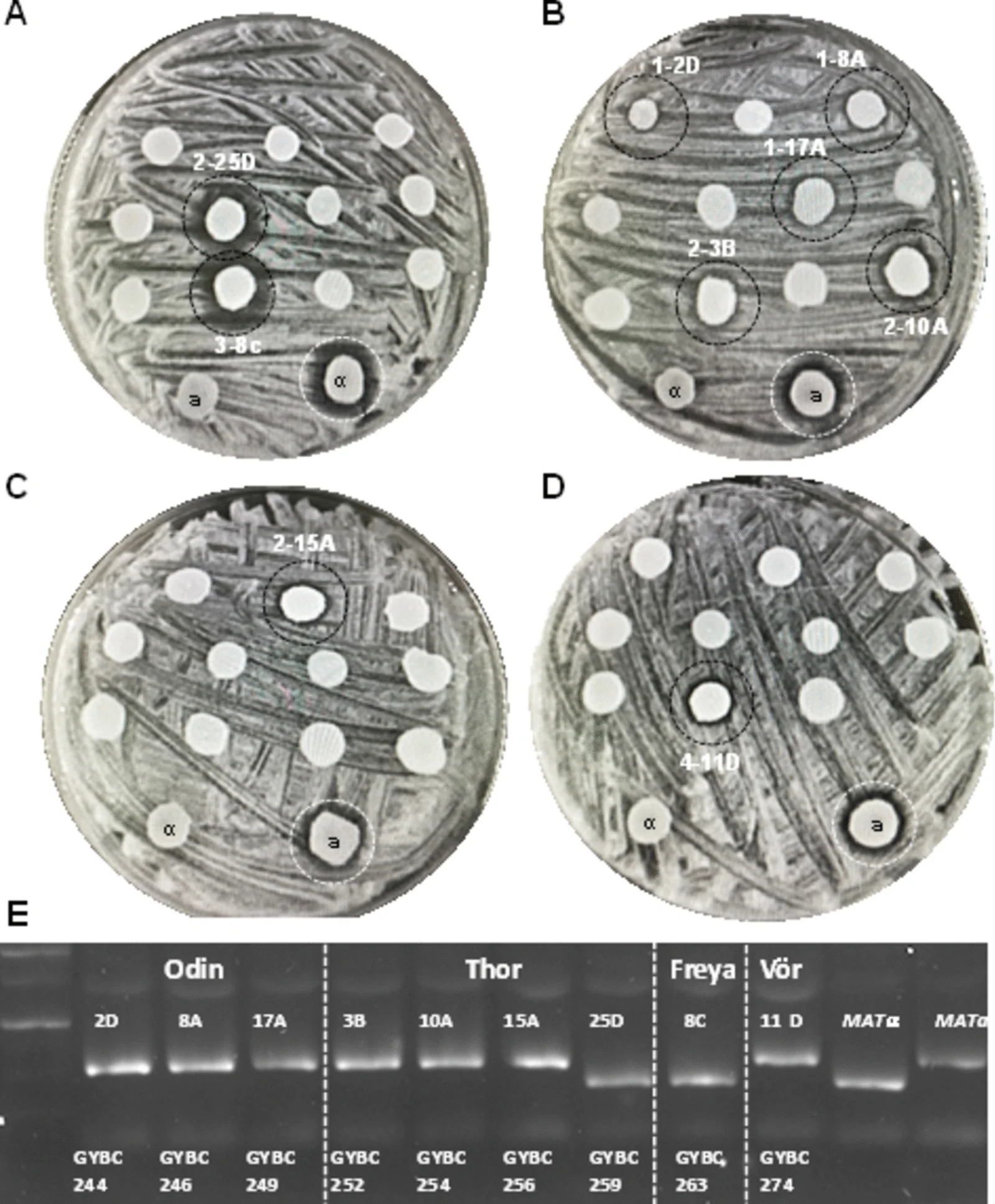
Isolation of heterothallic Kveik spore clones. **A–D** Four halo assay plates inoculated with 10–11 query strains each are shown. Query strains that induced halos are encircled. Strain assignments were as follows: 1, Odin; 2, Thor; 3, Freya; and 4, Vör. Isolate numbers correspond to samples in **E**. **E** Gel image of mating type PCR reactions assigning either *MAT****a*** or *MATα* to the spore clones (also indicated by their GYBC number, see Table 1). *MAT****a*** and *MATα* bands (right lanes) were used as controls; size marker in the left lane. Note: halo assay results and *MAT*-PCR diagnostics confirm genotype-matched phenotype in all strains

Recent studies on Norwegian farmhouse *S. cerevisiae* ale yeasts, also known as Kveik yeasts, indicated superior sporulation and germination properties of these yeasts compared to other ale yeast strains (Preiss et al. 2018; Dippel et al. 2022). The sporulation efficiency of the four Kveik strains was in the range of 31–78% (Dippel et al. 2022). This led us to investigate the breeding potential of these yeast strains. Therefore, we sporulated the four Kveik yeast strains and micromanipulated 25 four-spored asci for each strain, which led to 36–64 spore clones per strain. Spore clones thereof were queried for their mating abilities in halo assays. Surprisingly, already in a small selection of spore clones, we were able to identify a number of strains not only with the ability to mate as *MAT****a*** but also with the ability to mate as *MATα* strains (Fig. 3A–D). For Kveik 1, we obtained three **a**-maters out of eight colonies tested, for Kveik 2 three **a**-maters and one α-mater out of eight tested colonies, for Kveik 3 one α-mater out of 13 colonies and for Kveik 4 one **a**-mater out of two colonies. We analyzed the composition of the *MAT*-loci of these nine stable mating strains and found that the strains’ genotypes matched their phenotypic mating abilities (Fig. 3E). The established heterothallic lines were named Odin, Thor, Freya and Vör based on Nordic mythology to appraise their origin from Kveik strains Kveik 1, Kveik 2, Kveik 3 and Kveik 4, respectively (see Table 1).

### Analysis of the HO genes of spore clones identified inactivating mutations

**Fig. 4:**
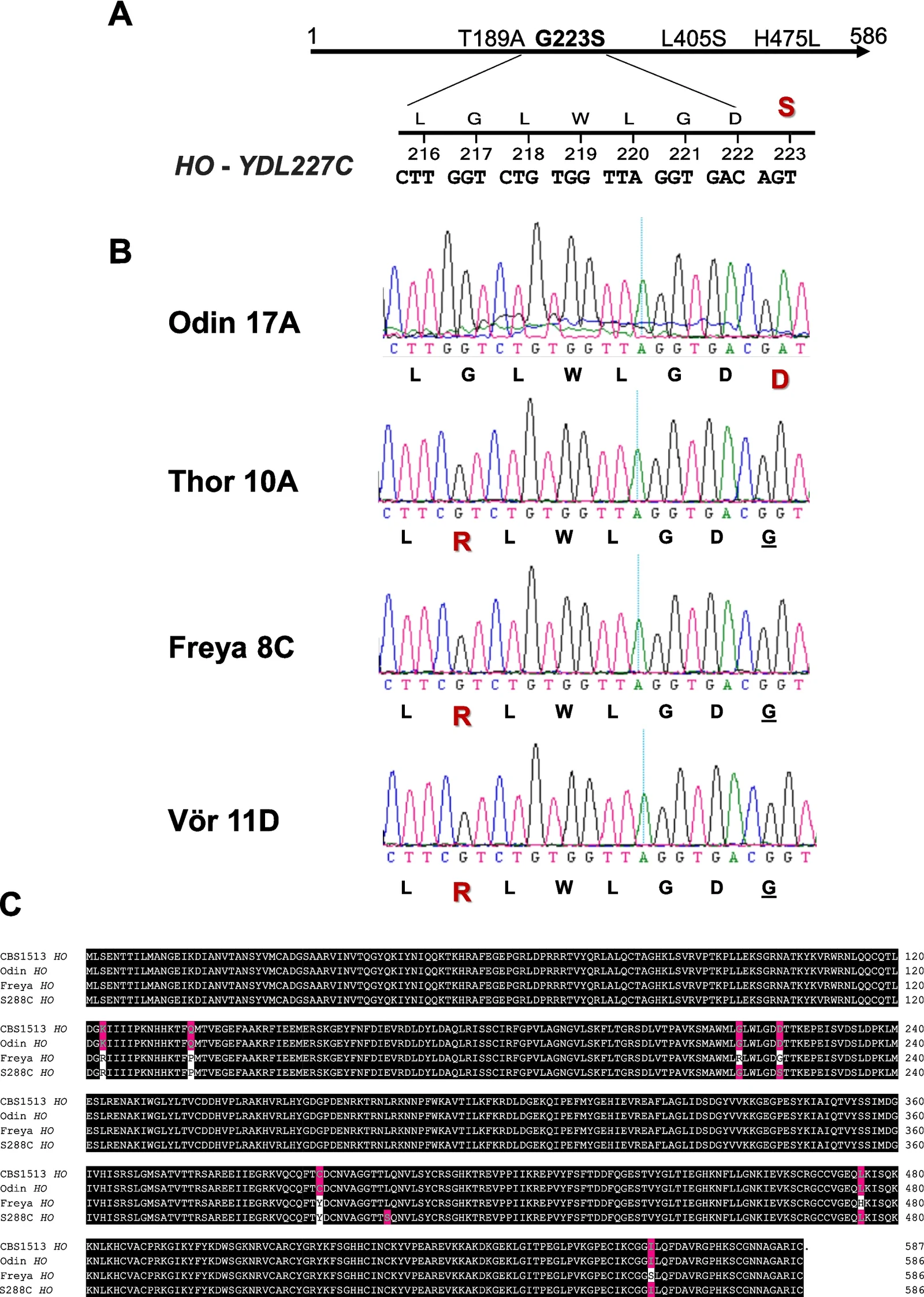
Analysis of *HO* gene sequences of heterothallic strains. **A** The S288C *HO* allele is shown with its mutations resulting in amino acid changes. The G223S amino acid exchange, due to a G > A transition, abolishes the Ho function in this strain. **B** Sequence analyses of the *HO* genes of the indicated heterothallic Kveik yeasts. Chromatograms are shown covering the region 216–223 of the Ho protein. Mutations resulting in amino acid changes are highlighted. **C** Alignment of Ho protein sequences of *S. carlsbergensis* (CBS 1513), Odin, Freya (identical to Thor and Vör) and S288C. Matching residues are shaded in black. Residues differing from Freya are shaded with solid deep magenta. Note: Odin’s Ho sequence matches that of lager yeast. The alignment was generated with DNASTAR MegAlign V12.1.0. Ho proteins of S288C and CBS 1513 were obtained from accession numbers NM_001180287 and AZCJ01000000, respectively

Strains exhibiting a stable mating type are either heterothallic or bear a deletion at the *MAT*-locus and thus mate as **a**-cells by default (Klar 1980). Heterothallic *Saccharomyces* strains cannot switch their mating type due to a defect in the *HO* gene (Haber 2012). *HO* codes for an endonuclease that initiates the gene conversion underlying a mating type switch by inducing a double-strand break at the *MAT* locus (Fig. 1B). Laboratory strains, e.g. the BY-series of strains, are heterothallic due to mutations in the *HO*-gene leading to four amino acid changes—T189A, G223S, L405S and H475L of which the G223S exchange abolishes Ho function (Ekino et al. 1999; Fig. 4A). To investigate whether the stable mating types of our strains are due to mutations in *HO*, we sequenced the *HO*-alleles of Odin 17A, Thor 10A, Freya 8C and Vör 11D (Fig. 4B). The *HO* sequences of Thor, Freya and Vör were identical but differed from Odin’s. Odin’s *HO* sequence showed a G to A mutation at position 668 also leading to an amino acid substitution at amino acid position 223 as in the S288C sequence. Instead of the S288C G223S exchange, Odin revealed a G223D mutation. The G233D exchange is expected to abolish the Ho function as well. This mutation is also present in the haploid heterothallic laboratory *S. cerevisiae* strain D273-10B, *MATα* and in other isolates (Sherman 1963; Song et al. 2015; Katz Etzov et al. 2010). Thor, Freya and Vör were wildtypes at position 223; however, they all harboured a C to G mutation at position 649 resulting in a G217R mutation. This glycine residue at position 217 is conserved in the protein family of homing endonucleases (pfam05203), which suggests that this position is also critical for Ho function (see below). The alignment of Ho protein sequences further revealed that Odin’s Ho protein sequence—and also its *HO* ORF (open reading frame) sequence—is identical to the *S. cerevisiae* Ho proteins (and *HO* ORFs) in Saaz and Frohberg lager yeasts (Fig. 4C, Supplemental Fig. S1). Overall, Odin 17A presented a known deleterious mutation in *HO* that, surprisingly, is also present in all lager yeast strains, while Thor, Freya and Vör revealed a novel mutation (resulting in a G217R exchange) in their *HO* sequences.

### Analysis of budding patterns in heterothallic strains

**Fig. 5:**
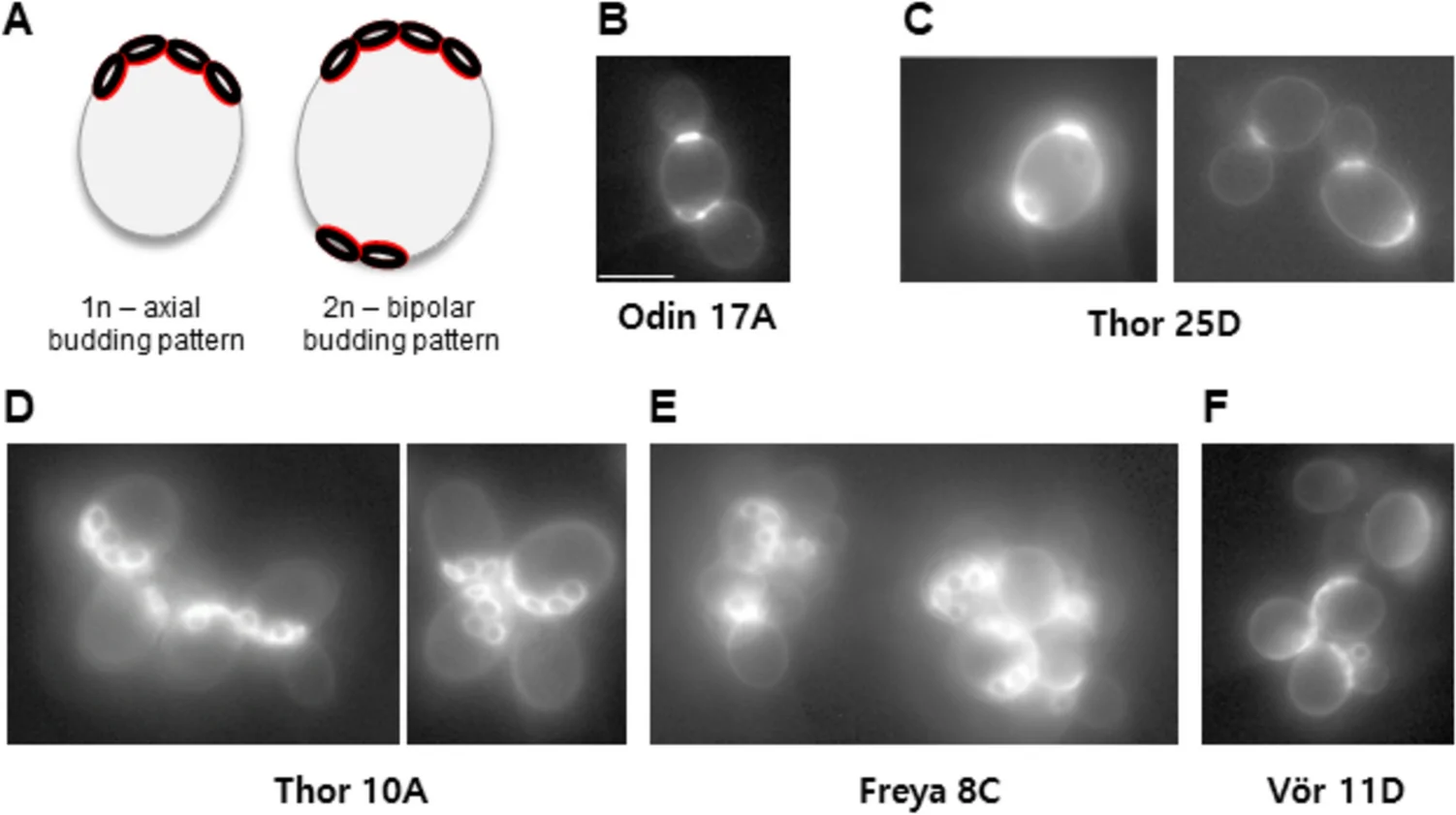
Budding patterns in heterothallic strains. **A** Schematic drawing of haploid and diploid *S. cerevisiae* cells highlighting the position of chitin rings/bud scars as remnants of previous rounds of cell division. Budding in haploid cells is restricted to one pole (the proximal pole with which the cell was connected to its mother), while bud site selection in diploid cells can utilize both poles. **B–F** Representative fluorescence microscopy images of calcofluor-stained cells of the indicated strains clearly distinguish **D–F** axial and **B**, **C** bipolar budding patterns

Haploid *S. cerevisiae MAT****a*** or *MATα* cells generate daughter cells only at the proximal pole with which they were connected to their mother cell. This is known as the axial budding pattern in contrast to bipolar budding in diploid *MAT****a/****α* cells, in which budding may occur at both cell poles (Freifelder 1960; Chant and Pringle 1995). This indicates that the budding pattern in *S. cerevisiae* is determined by cell type rather than ploidy (Lord et al. 2002). Hence, budding pattern analysis does not unequivocally indicate the ploidy of the strains Freya and Vör may either be haploid *MATα* or *MAT****a*** cells but could also be diploid *MATα/α* or *MAT****a****/****a*** cells, respectively. Nevertheless, we analyzed budding patterns in Kveik spore clones to gain further insight into their physiology. Observation of calcofluor stained cells indicated that the heterothallic strains Thor 10A, Freya 8C and Vör 11D budded in an axial manner indicating that their behaviour matched that expected of a haploid strain. However, Odin 17A and Thor 25D, unexpectedly, showed bipolar budding (Fig. 5, see ‘Discussion’ section).

### Breeding of heterothallic Kveik strains with haploid tester strains

**Fig. 6:**
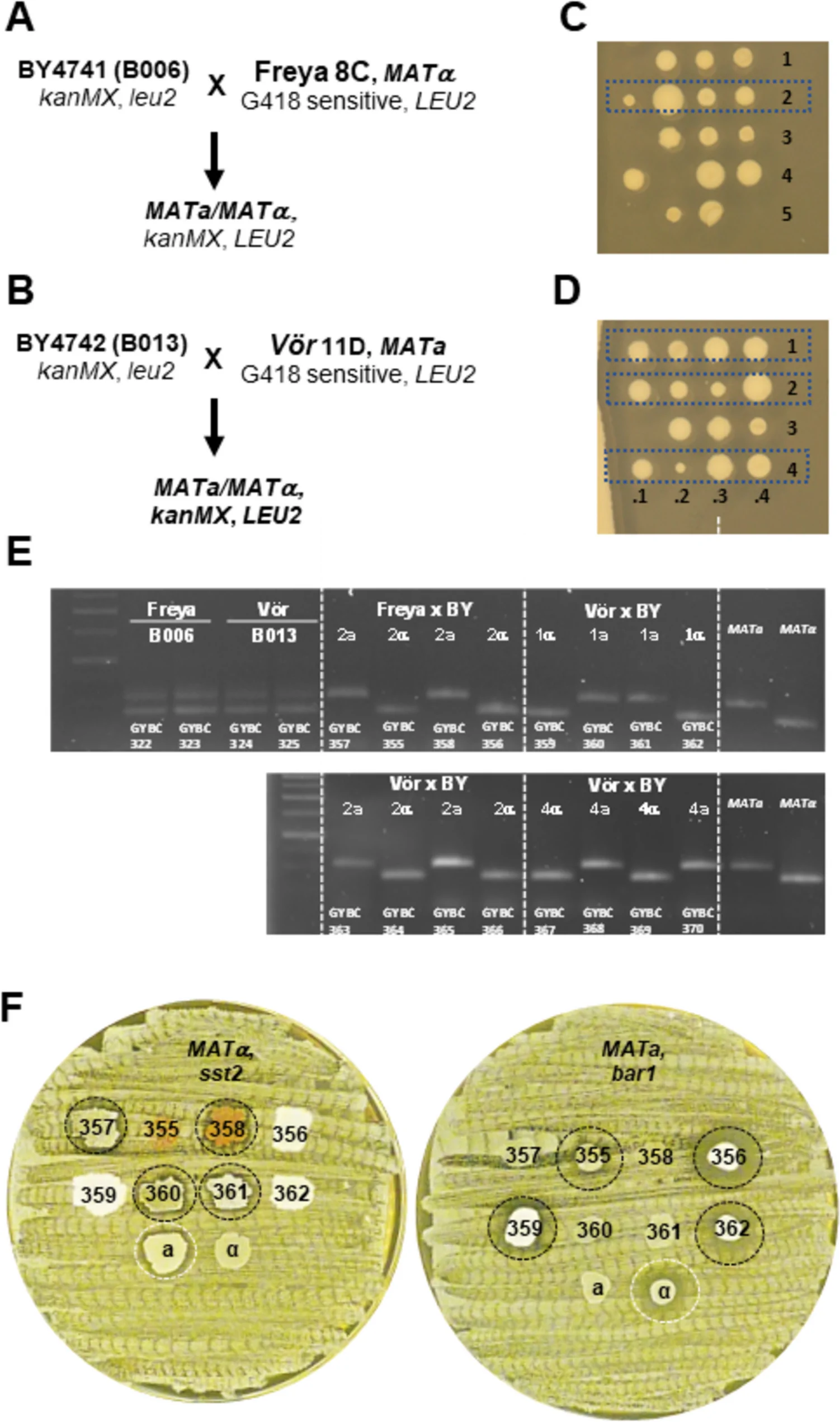
Breeding of heterothallic strains with haploid tester strains. **A**, **B** Freya and Vör were selected as *MATα* and *MAT****a*** strains and mated with compatible BY-strains using marker-assisted breeding and selection of zygotes. **C**, **D** Zygotes (Freya × B006 for dissection plate **C**; Vör × B013 for panel **D**) were sporulated and tetrads were dissected via micromanipulation. Four tetrads are numbered. **E** Mating type PCR diagnostics of zygotes and spore clones from complete tetrads. While zygotes showed both mating types as expected, tetrads of the crosses yielded a 2:2 segregation of *MAT****a*** and *MAT*α. *MAT****a*** and *MATα* bands (right lanes in each panel) were used as controls; size marker in the left lanes of each panel. Spore clones are also indicated with their GYBC collection number. **F** Halo assays of spore clones of one tetrad each derived from the Freya × B006 and Vör × B013 crosses. Strains that elicited a halo in the tester strain background are encircled, and strain identifiers are listed

To investigate if Freya and Vör can generate viable meiotic offspring with 1n laboratory strains, we crossed Freya 8C and Vör 11D with compatible haploid strains bearing complementing markers (Fig. 6A and B). Resulting zygotes were sporulated and tetrads were dissected by micromanipulation. All crosses yielded viable spores (Fig. 6C and D). Colonies derived from single tetrads (marked in Fig. 6C and D) were analyzed for their mating type by PCR and a 2:2 segregation of *MAT****a*** and *MATα* was observed for tetrads of both crosses (Fig. 6E). We tested the colonies derived from tetrads of both crosses for their phenotypic mating behaviour in halo assay. This indicated that their mating ability corresponded with their genotype at the *MAT* locus (Fig. 6F). This outcome indicated that Freya and Vör are haploid strains. It further indicated that the *HO* gene of Freya and Vör is non-functional as all colonies were heterothallic.

### Analysis of the *PAD1* and *FDC1* genes

A clear distinction between ale and lager yeasts is the ability of ale yeasts to produce the clove-like aroma compound 4-vinyl guaiacol (4-VG), which is regarded as an off-flavor in lager beer. Therefore, ale yeasts are mostly POF + and lager yeasts are POF-. Lager yeasts bear mutations in either *PAD1* or *FDC1* coding for ferulic acid decarboxylase (Mukai et al. 2014; Gallone et al. 2016). Kveik yeasts were originally described as POF-; however, this was recently revisited (Preiss et al. 2018, 2024). To gain insight into the potential of our strains to generate 4-VG from ferulic acid, we sequenced the *PAD1* and *FDC1* genes of the heterothallic Kveik strains (Supplemental Fig. S2–S5). Thor, Freya and Vör harboured wildtype *PAD1* and *FDC1* sequences and are thus regarded as POF + . Odin, however, revealed a common nonsense mutation in *PAD1* at position 305 (TTG > TAG). All four strains differed from S288C *PAD1* in two positions (C112T and C140T) resulting in the amino acid changes H38Y and A47V, which is common amongst ale yeasts (Mukai et al. 2014). Odin’s *PAD1* sequence, however, was more similar to S288C *PAD1* sharing five SNPs (Supplemental Fig. S2 and S3). Odin’s *FDC1* sequence differed from S288C *FDC1* in six positions. Importantly, one frameshift mutation at position 502 inserted an additional A residue, which led to a stop codon of three codons downstream (Supplemental Fig. S4 and S5). This frameshift mutation is also found in the lager yeast WS34/70. Together, this indicates that Odin has deleterious mutations in both *PAD1* and *FDC1* and is, therefore, regarded as POF-.

### Fermentation analyses with the Kveik spore clones

**Fig. 7:**
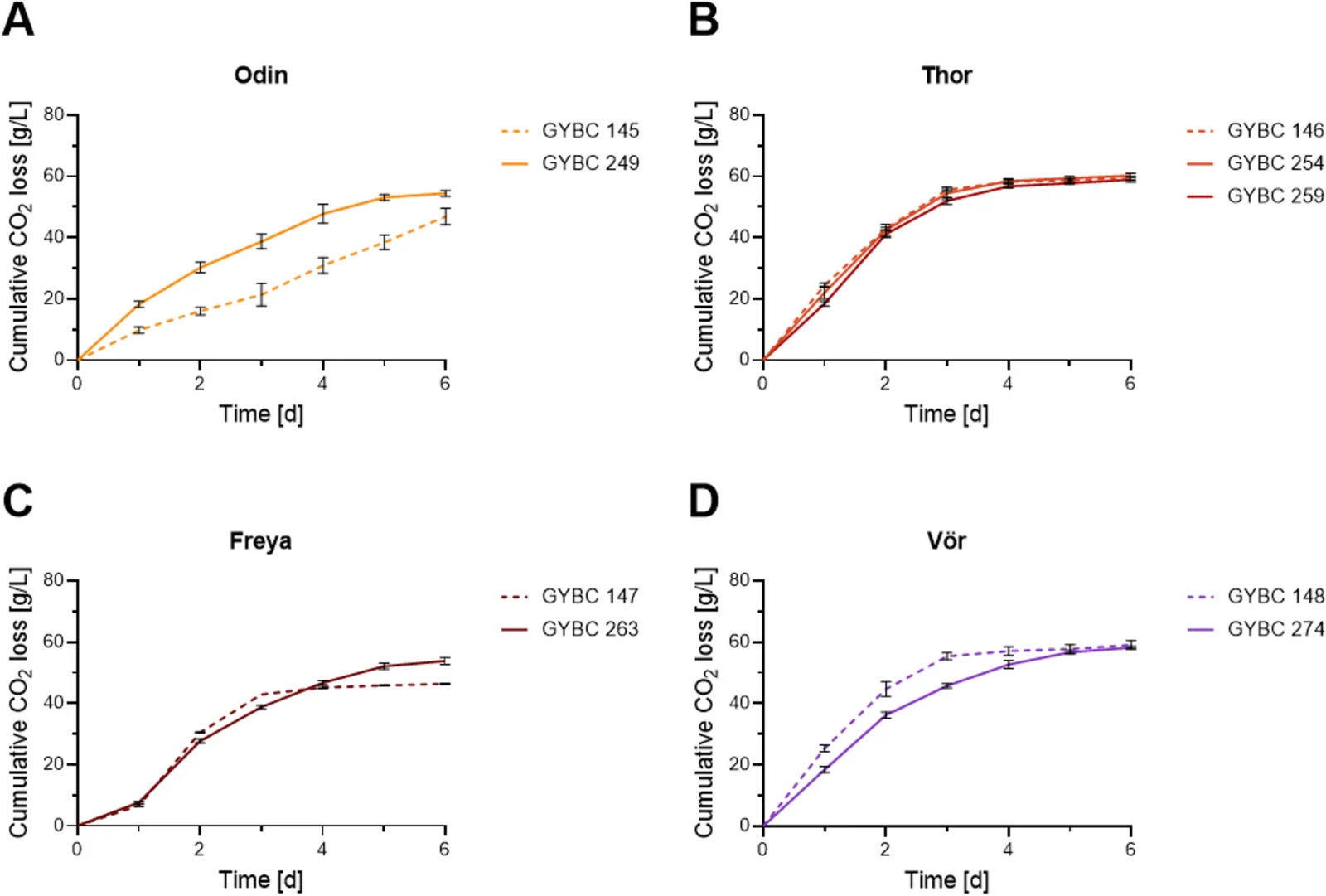
Fermentation analyses of heterothallic strains. **A–D** Fermentation curves of the indicated heterothallic Kveik strains (solid lines) compared to their parental strains (dashed lines). Curves are based on the cumulative loss of CO_2_ measured in daily intervals. Fermentations were carried out in triplicate. Bars indicate a standard deviation of the mean

The heterothallic strains were compared to their parental strains in lab-scale fermentations (Fig. 7 and Table 3). The fermentation curves show an equal behaviour of both heterothallic Kveik strains Thor 10A and Thor 25D fermenting 15° Plato wort compared to their parental strain (Fig. 7B) while consuming less glucose leading to lower ethanol levels than the parental strain (Table 3). Compared to its parental strain, Odin revealed a faster increase in cumulative CO_2_ mass loss (Fig. 7A) but no significant difference in sugar consumption or ethanol production (Table 3). A slower increase in the fermentation curve can be seen for Vör (Fig. 7D) reaching the same level of cumulative CO_2_ mass loss as the parental strain, but three days later. Similar to the Thor strains, Vör also produced less ethanol while consuming less sugars than its parental strain (Table 3). In addition to a lower glucose consumption, higher residual maltose and maltotriose levels were measured in the fermentation supernatant of wort fermented using Vör. Among the Kveik spore clones tested in lab-scale fermentations, Freya showed improved characteristics concerning sugar utilization and ethanol production compared to the parental strain, which was not able to consume all the maltotriose of the wort as reported earlier (Dippel et. al. 2022). Next to higher maltotriose utilization Freya also produced increased ethanol levels (Table 3).

**Table 3:** Residual sugars and total ethanol content at the end of fermentation

	Strain	Glucose (g/L)	Maltose (g/L)	Maltotriose (g/L)	Ethanol (g/L)	Ethanol (%)
Odin	GYBC 145	0.03 ± 0.03	0.52 ± 0.05	0.48 ± 0.05	53.07 ± 1.14	6.72 ± 0.12
	GYBC 249	0.04 ± 0.01	0.46 ± 0.04	0.49 ± 0.03	53.24 ± 0.64	6.75 ± 0.06
Thor 10A Thor 25D	GYBC 146	0.07 ± 0.03	0.69 ± 0.07	0.61 ± 0.04	58.48 ± 0.31	7.41 ± 0.00
	GYBC 254	0.14 ± 0.02*	0.77 ± 0.05	0.66 ± 0.03	57.53 ± 0.40*	7.29 ± 0.06
	GYBC 259	0.14 ± 0.02*	0.77 ± 0.01	0.63 ± 0.03	56.88 ± 0.21*	7.21 ± 0.00
Freya	GYBC 147	0.16 ± 0.02	0.73 ± 0.13	5.77 ± 1.01	48.94 ± 0.87	6.20 ± 0.10
	GYBC 263	0.18 ± 0.05	0.83 ± 0.10	1.16 ± 0.05*	58.25 ± 1.10*	7.38 ± 0.12*
Vör	GYBC 148	0.07 ± 0.01	0.67 ± 0.03	0.63 ± 0.01	57.33 ± 0.31	7.26 ± 0.06
	GYBC 274	0.16 ± 0.03*	0.88 ± 0.01*	0.74 ± 0.01*	54.71 ± 0.20*	6.93 ± 0.06*

Results are presented as the mean ± SD of three biological replicates. Significant differences in sugar degradation and ethanol formation between the spore clones and their respective parental strains were determined using a two-tailed unpaired *t*-test with Welch’s correction, *p < 0.05 as compared to the control (respective parental strain)

## Discussion

There is a strong trend to diversify beer styles, promoted by craft breweries and a desire for new aromas to meet consumer preferences and also embrace necessary adaptations to mitigate climate change-associated effects (Peris et al. 2018). European Farmhouse yeasts and particularly Norwegian Kveik yeasts offer traditional beer yeasts with interesting fermentation traits that have a long been used locally but are distinct from mainstream brewer’s yeasts (Foster et al. 2022; Preiss et al. 2024). Here, we extended previous work on Kveik yeasts and elucidated their potential for conventional yeast breeding.

Kveik yeasts have been placed in a sister group to the Beer 1/ale yeast group and were described as being aneuploid/tetraploid (Gallone et al. 2016; Preiss et al. 2018, 2024). However, these strains produced viable spores allowing breeding approaches (Preiss et al. 2018; Dippel et al. 2022). Breeding of different strains is highly facilitated by using heterothallic strains with stable mating types. Here, we show that, surprisingly, such strains can be derived from Kveiks. A key gene required for the gene conversion event governing homothallism in *Saccharomyces* is the *HO* gene (Haber 1998). Analyses of *HO* sequences of heterothallic Kveik strains revealed mutations in a conserved domain of homing endonucleases. While we found a novel inactivating mutation in the Ho protein (G217R) of three Kveik strains, we noted that Odin’s *HO* gene sequence is identical to that found in Saaz and Frohberg lager yeasts, suggesting a shared origin. This encompasses eight nucleotide positions, four of which are also shared by S288C (Supplementary Fig. S1). Interestingly, the presence of recessive *S. cerevisiae* mutant *HO* alleles in lager yeasts may, actually, have been the cause that allowed the rare isolation of mating-competent lager yeast spore clones. Another recessive mutation in Kveik strains may govern the budding pattern of the derived spore clones. While several strains showed the expected axial budding pattern of haploid strains, the cause for bipolar budding in Odin 17A and Thor 25D is currently unknown. Budding patterns in wildtype *S. cerevisiae* are determined by cell type and the bipolar budding pattern occurs only in *MAT****a/****α* strains (Chant and Herskowitz 1991). In *S. cerevisiae*, four genes have been described to be required for axial budding, *AXL1*, *AXL2*, *BUD3* and *BUD4*, and mutations in any one of these genes convert axial budding to bipolar budding (Lord et al. 2002). This suggests that Odin 17A and Thor 25D may harbour inactivating mutation(s) in (at least) one of these genes. This is under current investigation.

Heterothallic haploid lager yeast spore clones could in principle facilitate at least one round of conventional yeast breeding to evolve these strains as has been demonstrated previously (Garcia Sanchez et al. 2012; Gorter de Vries et al. 2019).

Kveik strains are apparently heterozygous for *HO* as spore clones with *MAT****a****/α* genotype were also obtained with a non-mater phenotype. Determination of the underlying ploidy of Freya and Vör spore clones revealed that these strains are haploid. Viable spores derived from crosses with Freya and Vör behaved consistently like haploids in further crosses (our unpublished results). This indicates that with these heterothallic Kveik strains, the full force of the ‘awesome power of yeast genetics’ can be employed for improvement and development of novel yeasts to benefit a wide range of fermentations.

Similarly, haploid cell lines in other systems were instrumental to open new research avenues, e.g. in human haploid cell lines, in the ‘obligate diploid’ human fungal pathogen *Candida albicans* or in solopathogenic haploid cell lines of the plant pathogen *Ustilago maydis*, besides making *S. cerevisiae* the ‘model eukaryote’ in the first place (Botstein and Fink 1988, 2011; Bölker et al. 1995; Hickman et al. 2013; Essletzbichler et al. 2014; Segal et al. 2018).

Conventional yeast breeding may now allow to cross Kveik beer yeasts with other *Saccharomyces* species, particularly *Saccharomyces kudriavzevii* and *Saccharomyces uvarum* to access the *Saccharomyces* biodiversity found, e.g. in apple and cider yeasts to contribute to strain improvements. Natural hybrids between *S. cerevisiae* and *S. uvarum* or *S. cerevisiae* and *S. kudriavzevii* have already been used successfully in wine fermentations (Gonzalez et al. 2006; Le Jeune et al. 2007; Lopes et al. 2010; Peris et al. 2012; Perez et al. 2022; Winans 2022). Such hybrids generated lower amounts of ethanol by increased synthesis of glycerol and increased levels of higher alcohols (Origone et al. 2018).

Initially, Kveik yeasts were described as being POF- (Preiss et al. 2018). Recently, however, it was found that not all Kveik strains are POF- (Preiss et al. 2024). Our data indicate that of the strains used in this study, only Odin is POF- while Thor, Freya and Vör are POF + . However, we did not look into the potential heterozygosity of inactivating mutations in the parental Kveik strains. The haploid heterothallic brewer’s yeasts can now be subjected to laboratory evolution, mutation and selection and other breeding programs to analyze complex traits and to yield non-GMO (non-genetically modified organism) derivative strains and backcrossed strains. This allows, for example, to rethink lager yeast, i.e. develop a lager yeast without any *S. eubayanus* contribution. Thus, Odin may represent a starter yeast for lager beer, while Freya may represent a POF + yeasts ideally suited for ale, wheat beer and wine fermentations.

In summary, we isolated and characterized haploid heterothallic brewer’s yeast strains with both *MAT****a*** and *MATα* mating types derived from European Farmhouse Kveik yeasts. These yeasts present fast fermenting, maltose and maltotriose-utilizing strains that provide novel technological benefits for the improvement of fermentation traits of interest to beer and wine fermentations, baking, or biotechnology.

## Supplementary Information

Below is the link to the electronic supplementary material.

Supplementary file1 (PDF 981 KB)

## Data Availability

All data supporting the findings of this study are available within the paper and its Supplementary Information. Sequences generated in this study were also deposited with GenBank under accession numbers PQ154469-PQ154480.
