# Supplementary material for: Isolation and characterization of haploid heterothallic beer yeasts
Source: Appl Microbiol Biotechnol. 2025 Jan 22;109(1):17. doi: 10.1007/s00253-024-13397-8 (PMC11754353; doi:10.1007/s00253-024-13397-8)
Supplement: Supplementary file 1 — Supplementary file1 (PDF 981 KB) [file 253_2024_13397_MOESM1_ESM.pdf]

Applied Microbiology and Biotechnology

Supplemental Material

Isolation and characterization of haploid heterothallic beer yeasts

Jennifer Badura[1,2], Beatrice Bernardi[1,2,§], Judith Muno-Bender[1,2], Katrin Matti[1,2], Kerstin Zimmer[1,2] and Jürgen Wendland[1,2*]

Department of Microbiology and Biochemistry, Hochschule Geisenheim University, Von-Lade-Strasse 1, 65366 Geisenheim, Germany

[2] Geisenheim Yeast Breeding Center, Hochschule Geisenheim University, Von-Lade-Strasse 1, 65366 Geisenheim

[§] current address: Formo Bio GmbH, Weißmüllerstraße 50 60314 Frankfurt am Main

Tel. +49 (0)6722 502 332

Mobil. +49 (0)178 31 64 267

Fax . +49 (0)6722 502 330
e-mail: Juergen.Wendland@hs-gm.de

```
         10 20 30 40 50 60 70 80 90 100 110 120
CBS1513  ATGCTTTCTGAAAACACGACTATTCTGATGGCTAACGGTGAAATTAAAGACATCGCAAACGTCACGGCTAACTCTTACGTTATGTGCGCAGATGGCTCCGCTGCCCGCGTCATAAATGTC 120
WS34-70  ATGCTTTCTGAAAACACGACTATTCTGATGGCTAACGGTGAAATTAAAGACATCGCAAACGTCACGGCTAACTCTTACGTTATGTGCGCAGATGGCTCCGCTGCCCGCGTCATAAATGTC 120
Odin     ATGCTTTCTGAAAACACGACTATTCTGATGGCTAACGGTGAAATTAAAGACATCGCAAACGTCACGGCTAACTCTTACGTTATGTGCGCAGATGGCTCCGCTGCCCGCGTCATAAATGTC 120
Thor     ATGCTTTCTGAAAACACGACTATTCTGATGGCTAACGGTGAAATTAAAGACATCGCAAACGTCACGGCTAACTCTTACGTTATGTGCGCAGATGGCTCCGCTGCCCGCGTCATAAATGTC 120
Freya    ATGCTTTCTGAAAACACGACTATTCTGATGGCTAACGGTGAAATTAAAGACATCGCAAACGTCACGGCTAACTCTTACGTTATGTGCGCAGATGGCTCCGCTGCCCGCGTCATAAATGTC 120
Vör      ATGCTTTCTGAAAACACGACTATTCTGATGGCTAACGGTGAAATTAAAGACATCGCAAACGTCACGGCTAACTCTTACGTTATGTGCGCAGATGGCTCCGCTGCCCGCGTCATAAATGTC 120
S288C    ATGCTTTCTGAAAACACGACTATTCTGATGGCTAACGGTGAAATTAAAGACATCGCAAACGTCACGGCTAACTCTTACGTTATGTGCGCAGATGGCTCCGCTGCCCGCGTCATAAATGTC 120

         130 140 150 160 170 180 190 200 210 220 230 240
CBS1513  ACACAGGGCTATCAGAAAATCTATAATATACAGCAAAAAACCAAACACAGAGCTTTTGAAGGTGAACCTGGTAGGTTAGATCCCAGGCGTAGAACAGTTTATCAGCGTCTTGCATTACAA 240
WS34-70  ACACAGGGCTATCAGAAAATCTATAATATACAGCAAAAAACCAAACACAGAGCTTTTGAAGGTGAACCTGGTAGGTTAGATCCCAGGCGTAGAACAGTTTATCAGCGTCTTGCATTACAA 240
Odin     ACACAGGGCTATCAGAAAATCTATAATATACAGCAAAAAACCAAACACAGAGCTTTTGAAGGTGAACCTGGTAGGTTAGATCCCAGGCGTAGAACAGTTTATCAGCGTCTTGCATTACAA 240
Thor     ACACAGGGCTATCAGAAAATCTATAATATACAGCAAAAAACCAAACACAGAGCTTTTGAAGGTGAACCTGGTAGGTTAGATCCCAGGCGTAGAACAGTTTATCAGCGTCTTGCATTACAA 240
Freya    ACACAGGGCTATCAGAAAATCTATAATATACAGCAAAAAACCAAACACAGAGCTTTTGAAGGTGAACCTGGTAGGTTAGATCCCAGGCGTAGAACAGTTTATCAGCGTCTTGCATTACAA 240
Vör      ACACAGGGCTATCAGAAAATCTATAATATACAGCAAAAAACCAAACACAGAGCTTTTGAAGGTGAACCTGGTAGGTTAGATCCCAGGCGTAGAACAGTTTATCAGCGTCTTGCATTACAA 240
S288C    ACACAGGGCTATCAGAAAATCTATAATATACAGCAAAAAACCAAACACAGAGCTTTTGAAGGTGAACCTGGTAGGTTAGATCCCAGGCGTAGAACAGTTTATCAGCGTCTTGCATTACAA 240

         250 260 270 280 290 300 310 320 330 340 350 360
CBS1513  TGTACTGCAGGTCATAAATTGTCAGTCAGGGTCCCTACCAAACCACTGTTGGAAAAAAGTGGTAGAAATGCCACCAAATATAAAGTGAGATGGAGAAATCTGCAGCAATGTCAGACGCTT 360
WS34-70  TGTACTGCAGGTCATAAATTGTCAGTCAGGGTCCCTACCAAACCACTGTTGGAAAAAAGTGGTAGAAATGCCACCAAATATAAAGTGAGATGGAGAAATCTGCAGCAATGTCAGACGCTT 360
Odin     TGTACTGCAGGTCATAAATTGTCAGTCAGGGTCCCTACCAAACCACTGTTGGAAAAAAGTGGTAGAAATGCCACCAAATATAAAGTGAGATGGAGAAATCTGCAGCAATGTCAGACGCTT 360
Thor     TGTACTGCAGGTCATAAATTGTCAGTCAGGGTCCCTACCAAACCACTGTTGGAAAAAAGTGGTAGAAATGCCACCAAATATAAAGTGAGATGGAGAAATCTGCAGCAATGTCAGACGCTT 360
Freya    TGTACTGCAGGTCATAAATTGTCAGTCAGGGTCCCTACCAAACCACTGTTGGAAAAAAGTGGTAGAAATGCCACCAAATATAAAGTGAGATGGAGAAATCTGCAGCAATGTCAGACGCTT 360
Vör      TGTACTGCAGGTCATAAATTGTCAGTCAGGGTCCCTACCAAACCACTGTTGGAAAAAAGTGGTAGAAATGCCACCAAATATAAAGTGAGATGGAGAAATCTGCAGCAATGTCAGACGCTT 360
S288C    TGTACTGCAGGTCATAAATTGTCAGTCAGGGTCCCTACCAAACCACTGTTGGAAAAAAGTGGTAGAAATGCCACCAAATATAAAGTGAGATGGAGAAATCTGCAGCAATGTCAGACGCTT 360

         370 380 390 400 410 420 430 440 450 460 470 480
CBS1513  GATGGTAAGATAATAATAATTCCAAAAAACCATCATAAGACATTCCAAATGACAGTTGAAGGTGAGTTTGCCGCAAAACGCTTCATAGAAGAAATGGAGCGCTCTAAAGGAGAATATTTC 480
WS34-70  GATGGTAAGATAATAATAATTCCAAAAAACCATCATAAGACATTCCAAATGACAGTTGAAGGTGAGTTTGCCGCAAAACGCTTCATAGAAGAAATGGAGCGCTCTAAAGGAGAATATTTC 480
Odin     GATGGTAAGATAATAATAATTCCAAAAAACCATCATAAGACATTCCAAATGACAGTTGAAGGTGAGTTTGCCGCAAAACGCTTCATAGAAGAAATGGAGCGCTCTAAAGGAGAATATTTC 480
Thor     GATGGTAGGATAATAATAATTCCAAAAAACCATCATAAGACATTCCCAATGACAGTTGAAGGTGAGTTTGCCGCAAAACGCTTCATAGAAGAAATGGAGCGCTCTAAAGGAGAATATTTC 480
Freya    GATGGTAGGATAATAATAATTCCAAAAAACCATCATAAGACATTCCCAATGACAGTTGAAGGTGAGTTTGCCGCAAAACGCTTCATAGAAGAAATGGAGCGCTCTAAAGGAGAATATTTC 480
Vör      GATGGTAGGATAATAATAATTCCAAAAAACCATCATAAGACATTCCCAATGACAGTTGAAGGTGAGTTTGCCGCAAAACGCTTCATAGAAGAAATGGAGCGCTCTAAAGGAGAATATTTC 480
S288C    GATGGTAGGATAATAATAATTCCAAAAAACCATCATAAGACATTCCCAATGACAGTTGAAGGTGAGTTTGCCGCAAAACGCTTCATAGAAGAAATGGAGCGCTCTAAAGGAGAATATTTC 480

         490 500 510 520 530 540 550 560 570 580 590 600
CBS1513  AACTTTGACATTGAAGTTAGAGATTTGGATTATCTTGATGCTCAATTGAGAATTTCTAGCTGCATAAGATTTGGTCCAGTACTCGCAGGAAATGGTGTTTTATCTAAATTTCTCACTGGA 600
WS34-70  AACTTTGACATTGAAGTTAGAGATTTGGATTATCTTGATGCTCAATTGAGAATTTCTAGCTGCATAAGATTTGGTCCAGTACTCGCAGGAAATGGTGTTTTATCTAAATTTCTCACTGGA 600
Odin     AACTTTGACATTGAAGTTAGAGATTTGGATTATCTTGATGCTCAATTGAGAATTTCTAGCTGCATAAGATTTGGTCCAGTACTCGCAGGAAATGGTGTTTTATCTAAATTTCTCACTGGA 600
Thor     AACTTTGACATTGAAGTTAGAGATTTGGATTATCTTGATGCTCAATTGAGAATTTCTAGCTGCATAAGATTTGGTCCAGTACTCGCAGGAAATGGTGTTTTATCTAAATTTCTCACTGGA 600
Freya    AACTTTGACATTGAAGTTAGAGATTTGGATTATCTTGATGCTCAATTGAGAATTTCTAGCTGCATAAGATTTGGTCCAGTACTCGCAGGAAATGGTGTTTTATCTAAATTTCTCACTGGA 600
Vör      AACTTTGACATTGAAGTTAGAGATTTGGATTATCTTGATGCTCAATTGAGAATTTCTAGCTGCATAAGATTTGGTCCAGTACTCGCAGGAAATGGTGTTTTATCTAAATTTCTCACTGGA 600
S288C    AACTTTGACATTGAAGTTAGAGATTTGGATTATCTTGATGCTCAATTGAGAATTTCTAGCTGCATAAGATTTGGTCCAGTACTCGCAGGAAATGGTGTTTTATCTAAATTTCTCACTGGA 600

         610 620 630 640 650 660 670 680 690 700 710 720
CBS1513  CGTAGTGACCTTGTAACTCCTGCTGTAAAAAGTATGGCTTGGATGCTTGGTCTGTGGTTAGGTGACGATACAACAAAAGAGCCAGAAATATCAGTAGATAGCTTGGATCCTAAGCTAATG 720
WS34-70  CGTAGTGACCTTGTAACTCCTGCTGTAAAAAGTATGGCTTGGATGCTTGGTCTGTGGTTAGGTGACGATACAACAAAAGAGCCAGAAATATCAGTAGATAGCTTGGATCCTAAGCTAATG 720
Odin     CGTAGTGACCTTGTAACTCCTGCTGTAAAAAGTATGGCTTGGATGCTTGGTCTGTGGTTAGGTGACGATACAACAAAAGAGCCAGAAATATCAGTAGATAGCTTGGATCCTAAGCTAATG 720
Thor     CGTAGTGACCTTGTAACTCCTGCTGTAAAAAGTATGGCTTGGATGCTTCGTCTGTGGTTAGGTGACGGTACAACAAAAGAGCCAGAAATCTCAGTAGATAGCTTGGATCCTAAGCTAATG 720
Freya    CGTAGTGACCTTGTAACTCCTGCTGTAAAAAGTATGGCTTGGATGCTTCGTCTGTGGTTAGGTGACGGTACAACAAAAGAGCCAGAAATCTCAGTAGATAGCTTGGATCCTAAGCTAATG 720
Vör      CGTAGTGACCTTGTAACTCCTGCTGTAAAAAGTATGGCTTGGATGCTTCGTCTGTGGTTAGGTGACGGTACAACAAAAGAGCCAGAAATCTCAGTAGATAGCTTGGATCCTAAGCTAATG 720
S288C    CGTAGTGACCTTGTAACTCCTGCTGTAAAAAGTATGGCTTGGATGCTTGGTCTGTGGTTAGGTGACAGTACAACAAAAGAGCCAGAAATCTCAGTAGATAGCTTGGATCCTAAGCTAATG 720

         730 740 750 760 770 780 790 800 810 820 830 840
CBS1513  GAGAGTTTAAGAGAAAATGCGAAAATCTGGGGTCTCTACCTTACGGTTTGTGACGATCACGTTCCGCTACGTGCCAAACATGTAAGGCTTCATTATGGAGATGGTCCAGATGAAAACAGG 840
WS34-70  GAGAGTTTAAGAGAAAATGCGAAAATCTGGGGTCTCTACCTTACGGTTTGTGACGATCACGTTCCGCTACGTGCCAAACATGTAAGGCTTCATTATGGAGATGGTCCAGATGAAAACAGG 840
Odin     GAGAGTTTAAGAGAAAATGCGAAAATCTGGGGTCTCTACCTTACGGTTTGTGACGATCACGTTCCGCTACGTGCCAAACATGTAAGGCTTCATTATGGAGATGGTCCAGATGAAAACAGG 840
Thor     GAGAGTTTAAGAGAAAATGCGAAAATCTGGGGTCTCTACCTTACGGTTTGTGACGATCACGTTCCGCTACGTGCCAAACATGTAAGGCTTCATTATGGAGATGGTCCAGATGAAAACAGG 840
Freya    GAGAGTTTAAGAGAAAATGCGAAAATCTGGGGTCTCTACCTTACGGTTTGTGACGATCACGTTCCGCTACGTGCCAAACATGTAAGGCTTCATTATGGAGATGGTCCAGATGAAAACAGG 840
Vör      GAGAGTTTAAGAGAAAATGCGAAAATCTGGGGTCTCTACCTTACGGTTTGTGACGATCACGTTCCGCTACGTGCCAAACATGTAAGGCTTCATTATGGAGATGGTCCAGATGAAAACAGG 840
S288C    GAGAGTTTAAGAGAAAATGCGAAAATCTGGGGTCTCTACCTTACGGTTTGTGACGATCACGTTCCGCTACGTGCCAAACATGTAAGGCTTCATTATGGAGATGGTCCAGATGAAAACAGG 840

         850 860 870 880 890 900 910 920 930 940 950 960
CBS1513  AAGACAAGGAATTTGAGGAAAAATAATCCATTCTGGAAAGCTGTCACAATTTTAAAGTTTAAAAGGGATCTTGATGGAGAGAAGCAAATCCCTGAATTTATGTACGGCGAGCATATAGAA 960
WS34-70  AAGACAAGGAATTTGAGGAAAAATAATCCATTCTGGAAAGCTGTCACAATTTTAAAGTTTAAAAGGGATCTTGATGGAGAGAAGCAAATCCCTGAATTTATGTACGGCGAGCATATAGAA 960
Odin     AAGACAAGGAATTTGAGGAAAAATAATCCATTCTGGAAAGCTGTCACAATTTTAAAGTTTAAAAGGGATCTTGATGGAGAGAAGCAAATCCCTGAATTTATGTACGGCGAGCATATAGAA 960
Thor     AAGACAAGGAATTTGAGGAAAAATAATCCATTCTGGAAAGCTGTCACAATTTTAAAGTTTAAAAGGGATCTTGATGGAGAGAAGCAAATCCCTGAATTTATGTACGGCGAGCATATAGAA 960
Freya    AAGACAAGGAATTTGAGGAAAAATAATCCATTCTGGAAAGCTGTCACAATTTTAAAGTTTAAAAGGGATCTTGATGGAGAGAAGCAAATCCCTGAATTTATGTACGGCGAGCATATAGAA 960
Vör      AAGACAAGGAATTTGAGGAAAAATAATCCATTCTGGAAAGCTGTCACAATTTTAAAGTTTAAAAGGGATCTTGATGGAGAGAAGCAAATCCCTGAATTTATGTACGGCGAGCATATAGAA 960
S288C    AAGACAAGGAATTTGAGGAAAAATAATCCATTCTGGAAAGCTGTCACAATTTTAAAGTTTAAAAGGGATCTTGATGGAGAGAAGCAAATCCCTGAATTTATGTACGGCGAGCATATAGAA 960

         970 980 990 1000 1010 1020 1030 1040 1050 1060 1070 1080
CBS1513  GTTCGTGAAGCATTCTTAGCCGGCTTGATCGACTCAGATGGGTACGTTGTGAAAAAGGGCGAAGGCCCTGAATCTTATAAAATAGCAATTCAAACTGTTTATTCATCCATTATGGACGGA 1080
WS34-70  GTTCGTGAAGCATTCTTAGCCGGCTTGATCGACTCAGATGGGTACGTTGTGAAAAAGGGCGAAGGCCCTGAATCTTATAAAATAGCAATTCAAACTGTTTATTCATCCATTATGGACGGA 1080
Odin     GTTCGTGAAGCATTCTTAGCCGGCTTGATCGACTCAGATGGGTACGTTGTGAAAAAGGGCGAAGGCCCTGAATCTTATAAAATAGCAATTCAAACTGTTTATTCATCCATTATGGACGGA 1080
Thor     GTTCGTGAAGCATTCTTAGCCGGCTTGATCGACTCAGATGGGTACGTTGTGAAAAAGGGCGAAGGCCCTGAATCTTATAAAATAGCAATTCAAACTGTTTATTCATCCATTATGGACGGA 1080
Freya    GTTCGTGAAGCATTCTTAGCCGGCTTGATCGACTCAGATGGGTACGTTGTGAAAAAGGGCGAAGGCCCTGAATCTTATAAAATAGCAATTCAAACTGTTTATTCATCCATTATGGACGGA 1080
Vör      GTTCGTGAAGCATTCTTAGCCGGCTTGATCGACTCAGATGGGTACGTTGTGAAAAAGGGCGAAGGCCCTGAATCTTATAAAATAGCAATTCAAACTGTTTATTCATCCATTATGGACGGA 1080
S288C    GTTCGTGAAGCATTCTTAGCCGGCTTGATCGACTCAGATGGGTACGTTGTGAAAAAGGGCGAAGGCCCTGAATCTTATAAAATAGCAATTCAAACTGTTTATTCATCCATTATGGACGGA 1080
```

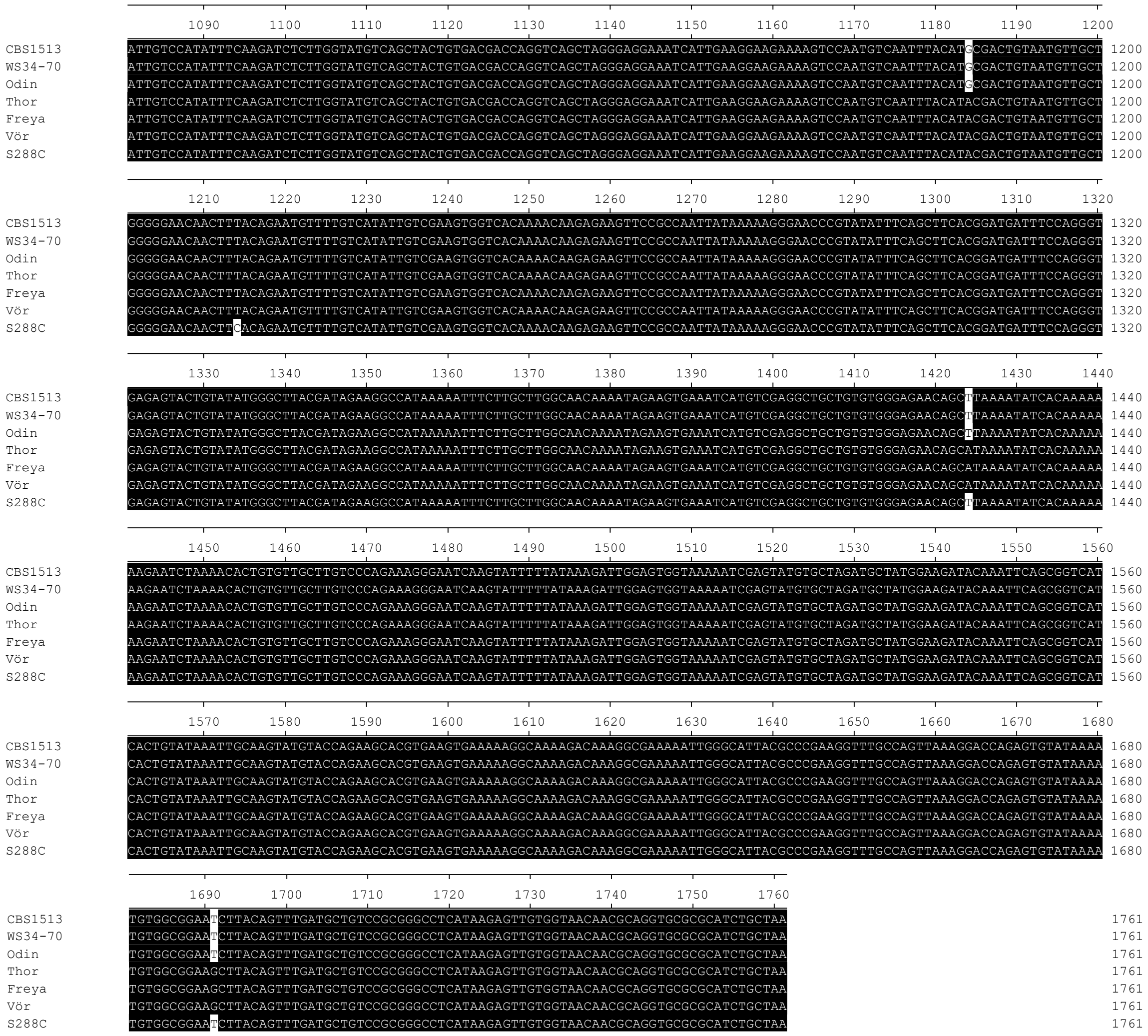

Figure S1 Alignment of *HO* ORF sequences. Sequences of the *HO* open reading frames of *S. carlsbergensis* (CBS 1513), Weihenstephan W34/70, Odin, Thor, Freya, Vör and S288C were aligned with DNASTAR MegAlign V12.1.0 (DNASTAR, Madison, WI, USA). Residues matching to Freya are shaded in black. Accession numbers of *HO* sequences: CBS 1513: AZCJ01000000 and WS34/70: AZAA01000000, Odin: PQ154469, Thor: PQ154470, Freya: PQ154471, Vör: PQ154472, S288C: NM_001180287.

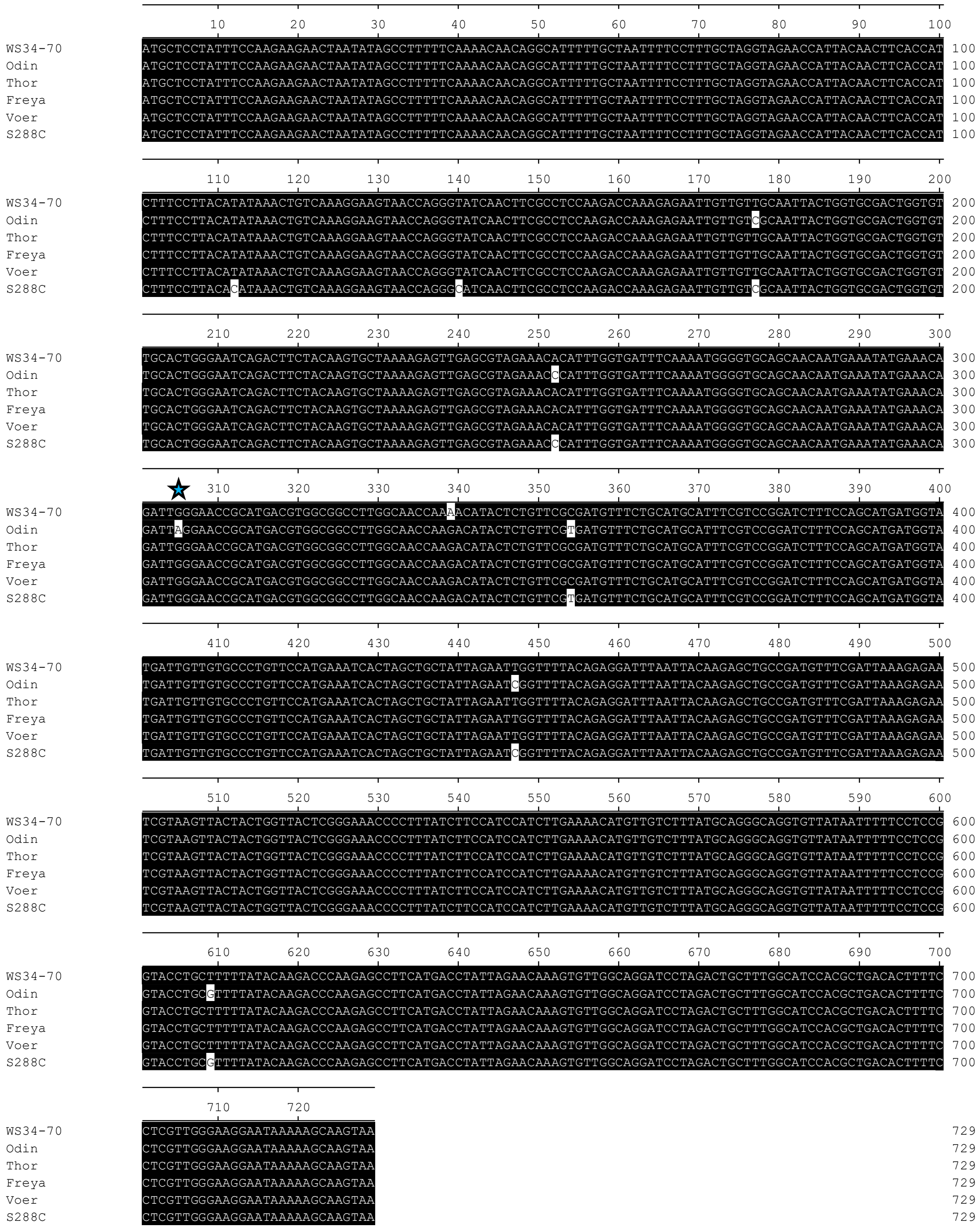

Figure S2 Alignment of *PAD1* ORF sequences. Sequences of the *PAD1* open reading frames (ORFs) of Weihenstephan W34/70, Odin, Thor, Freya, Vör and S288C were aligned with DNASTAR MegAlign V12.1.0 (DNASTAR, Madison, WI, USA). Residues matching to Freya are shaded in black. The TGG > TAA exchange leading to a nonsense codon in Odin's *PAD1* ORF sequence is marked by an asterisk. Accession numbers of *HO* sequences: WS34/70: AZAA01000000, Odin: PQ154473, Thor: PQ154474, Freya: PQ154475, Vör: PQ154476, S288C: BK006938.

```
                  10        20        30        40        50        60        70        80        90       100
WS34-70  MLLFPRRTNIAFFKTTGIFANFPLLGRTITTSPSFLTYKLSKEVTRVSTSPPRPKRIVVAITGATGVALGIRLLQVLKELSVETHLVISKWGAATMKYET 100
Odin     MLLFPRRTNIAFFKTTGIFANFPLLGRTITTSPSFLTYKLSKEVTRVSTSPPRPKRIVVAITGATGVALGIRLLQVLKELSVETHLVISKWGAATMKYET 100
Thor     MLLFPRRTNIAFFKTTGIFANFPLLGRTITTSPSFLTYKLSKEVTRVSTSPPRPKRIVVAITGATGVALGIRLLQVLKELSVETHLVISKWGAATMKYET 100
Freya    MLLFPRRTNIAFFKTTGIFANFPLLGRTITTSPSFLTYKLSKEVTRVSTSPPRPKRIVVAITGATGVALGIRLLQVLKELSVETHLVISKWGAATMKYET 100
Vör      MLLFPRRTNIAFFKTTGIFANFPLLGRTITTSPSFLTYKLSKEVTRVSTSPPRPKRIVVAITGATGVALGIRLLQVLKELSVETHLVISKWGAATMKYET 100
S288C    MLLFPRRTNIAFFKTTGIFANFPLLGRTITTSPSFLTHKLSKEVTRASTSPPRPKRIVVAITGATGVALGIRLLQVLKELSVETHLVISKWGAATMKYET 100

                 110       120       130       140       150       160       170       180       190       200
WS34-70  DWEPHDVAALATKTYSVRDVSACISSGSFQHDGMIVVPCSMKSLAAIRIGFTEDLITRAADVSIKENRKLLLVTRETPLSSIHLENMLSLCRAGVIIFPP 200
Odin     D.EPHDVAALATKTYSVRDVSACISSGSFQHDGMIVVPCSMKSLAAIRIGFTEDLITRAADVSIKENRKLLLVTRETPLSSIHLENMLSLCRAGVIIFPP 200
Thor     DWEPHDVAALATKTYSVRDVSACISSGSFQHDGMIVVPCSMKSLAAIRIGFTEDLITRAADVSIKENRKLLLVTRETPLSSIHLENMLSLCRAGVIIFPP 200
Freya    DWEPHDVAALATKTYSVRDVSACISSGSFQHDGMIVVPCSMKSLAAIRIGFTEDLITRAADVSIKENRKLLLVTRETPLSSIHLENMLSLCRAGVIIFPP 200
Vör      DWEPHDVAALATKTYSVRDVSACISSGSFQHDGMIVVPCSMKSLAAIRIGFTEDLITRAADVSIKENRKLLLVTRETPLSSIHLENMLSLCRAGVIIFPP 200
S288C    DWEPHDVAALATKTYSVRDVSACISSGSFQHDGMIVVPCSMKSLAAIRIGFTEDLITRAADVSIKENRKLLLVTRETPLSSIHLENMLSLCRAGVIIFPP 200

                 210       220       230       240
WS34-70  VPAFYTRPKSLHDLLEQSVGRILDCFGIHADTFPRWEGIKSK                                                           242
Odin     VPAFYTRPKSLHDLLEQSVGRILDCFGIHADTFPRWEGIKSK.                                                          243
Thor     VPAFYTRPKSLHDLLEQSVGRILDCFGIHADTFPRWEGIKSK.                                                          243
Freya    VPAFYTRPKSLHDLLEQSVGRILDCFGIHADTFPRWEGIKSK.                                                          243
Vör      VPAFYTRPKSLHDLLEQSVGRILDCFGIHADTFPRWEGIKSK.                                                          243
S288C    VPAFYTRPKSLHDLLEQSVGRILDCFGIHADTFPRWEGIKSK.                                                          243
```

Figure S3 Alignment of Pad1 protein sequences. Pad1 protein sequenced were translated from the ORF sequences shown in Figure S2. Residues matching to Freya are shaded in black. Note the stop codon in Odin’s Pad1 at position 102.

```
                  10        20        30        40        50        60        70        80        90       100
WS34-70  ATGAGGAAGCTAAATCCAGCTTTAGAATTTAGAGACTTTATCCAGGTCTTAAAAGATGAAGATGACTTAATCGAAATTACCGAAGAGATTGATCCAAATC 100
Odin     ATGAGGAAACTAAATCCAGCTTTAGAATTTAGAGACTTTATCCAGGTCTTAAAAGATGAAGATGACTTAATCGAAATTACCGAAGAGATTGATCCAAATC 100
Thor     ATGAGGAAGCTAAATCCAGCTTTAGAATTTAGAGACTTTATCCAGGTCTTAAAAGATGAAGATGACTTAATCGAAATTACCGAAGAGATTGATCCAAATC 100
Freya    ATGAGGAAGCTAAATCCAGCTTTAGAATTTAGAGACTTTATCCAGGTCTTAAAAGATGAAGATGACTTAATCGAAATTACCGAAGAGATTGATCCAAATC 100
Voer     ATGAGGAAGCTAAATCCAGCTTTAGAATTTAGAGACTTTATCCAGGTCTTAAAAGATGAAGATGACTTAATCGAAATTACCGAAGAGATTGATCCAAATC 100
S288C    ATGAGGAAGCTAAATCCAGCTTTAGAATTTAGAGACTTTATCCAGGTCTTAAAAGATGAAGATGACTTAATCGAAATTACCGAAGAGATTGATCCAAATC 100

                 110       120       130       140       150       160       170       180       190       200
WS34-70  TCGAAGTAGGTGCAATTATGAGGAAGGCCTATGAATCCCACTTACCAGCCCCGTTATTTAAAAATCTCAAAGGTGCTTCGAAGGATCTTTTCAGCATTTT 200
Odin     TCGAAGTAGGTGCAATTATGAGGAAGGCCTATGAATCCCACTTACCAGCCCCGTTATTTAAAAATCTCAAAGGTGCTTCGAAGGATCTTTTCAGCATTTT 200
Thor     TCGAAGTAGGTGCAATTATGAGGAAGGCCTATGAATCCCACTTACCAGCCCCGTTATTTAAAAATCTCAAAGGTGCTTCGAAGGATCTTTTCAGCATTTT 200
Freya    TCGAAGTAGGTGCAATTATGAGGAAGGCCTATGAATCCCACTTACCAGCCCCGTTATTTAAAAATCTCAAAGGTGCTTCGAAGGATCTTTTCAGCATTTT 200
Voer     TCGAAGTAGGTGCAATTATGAGGAAGGCCTATGAATCCCACTTACCAGCCCCGTTATTTAAAAATCTCAAAGGTGCTTCGAAGGATCTTTTCAGCATTTT 200
S288C    TCGAAGTAGGTGCAATTATGAGGAAGGCCTATGAATCCCACTTACCAGCCCCGTTATTTAAAAATCTCAAAGGTGCTTCGAAGGATCTTTTCAGCATTTT 200

                 210       220       230       240       250       260       270       280       290       300
WS34-70  AGGTTGCCCAGCCGGTTTAAGAAGTAAGGAGAAAGGAGATCATGGTAGAATTGCCCATCATCTGGGGCTCGACCCAAAAACAACTATCAAGGAAATCATA 300
Odin     AGGTTGCCCAGCCGGTTTAAGAAGTAAGGAGAAAGGAGATCATGGTAGAATTGCCCATCATCTGGGGCTCGACCCAAAAACAACTATCAAGGAAATCATA 300
Thor     AGGTTGCCCAGCCGGTTTAAGAAGTAAGGAGAAAGGAGATCATGGTAGAATTGCCCATCATCTGGGGCTCGACCCAAAAACAACTATCAAGGAAATCATA 300
Freya    AGGTTGCCCAGCCGGTTTAAGAAGTAAGGAGAAAGGAGATCATGGTAGAATTGCCCATCATCTGGGGCTCGACCCAAAAACAACTATCAAGGAAATCATA 300
Voer     AGGTTGCCCAGCCGGTTTAAGAAGTAAGGAGAAAGGAGATCATGGTAGAATTGCCCATCATCTGGGGCTCGACCCAAAAACAACTATCAAGGAAATCATA 300
S288C    AGGTTGCCCAGCCGGTTTGAGAAGTAAGGAGAAAGGAGATCATGGTAGAATTGCCCATCATCTGGGGCTCGACCCAAAAACAACTATCAAGGAAATCATA 300

                 310       320       330       340       350       360       370       380       390       400
WS34-70  GATTATTTGCTGGAGTGTAAGGAGAAGGAACCTCTCCCCCCAATCACTGTTCCTGTGTCATCTGCACCTTGTAAAACACATATACTTTCTGAAGAAAAAA 400
Odin     GATTATTTGCTGGAGTGTAAGGAGAAGGAACCTCTCCCTCCAATCACTGTTCCTGTGTCATCTGCACCTTGTAAAACACATATACTTTCTGAAGAAAAAA 400
Thor     GATTATTTGCTGGAGTGTAAGGAGAAGGAACCTCTCCCTCCAATCACTGTTCCTGTGTCATCTGCACCTTGTAAAACACATATACTTTCTGAAGAAAAAA 400
Freya    GATTATTTGCTGGAGTGTAAGGAGAAGGAACCTCTCCCTCCAATCACTGTTCCTGTGTCATCTGCACCTTGTAAAACACATATACTTTCTGAAGAAAAAA 400
Voer     GATTATTTGCTGGAGTGTAAGGAGAAGGAACCTCTCCCTCCAATCACTGTTCCTGTGTCATCTGCACCTTGTAAAACACATATACTTTCTGAAGAAAAAA 400
S288C    GATTATTTGCTGGAGTGTAAGGAGAAGGAACCTCTCCCCCCAATCACTGTTCCTGTGTCATCTGCACCTTGTAAAACACATATACTTTCTGAAGAAAAAA 400

                 410       420       430       440       450       460       470       480       490       500
WS34-70  TACATCTACAAAGCCTGCCAACACCATATCTATATGTTTCAGACGGTGGCAAGTACTTACAAACGTACGGAATGTGGATTCTTCAAACTCCAGATAAAAA 500
Odin     TACATCTACAAAGCCTGCCAACACCATATCTACATGTTTCAGACGGTGGCAAGTACTTACAAACGTACGGAATGTGGATTCTTCAAACTCCAGATAAAAA 500
Thor     TACATCTACAAAGCCTGCCAACACCATATCTACATGTTTCAGACGGTGGCAAGTACTTACAAACGTACGGAATGTGGATTCTTCAAACTCCAGATAAAAA 500
Freya    TACATCTACAAAGCCTGCCAACACCATATCTACATGTTTCAGACGGTGGCAAGTACTTACAAACGTACGGAATGTGGATTCTTCAAACTCCAGATAAAAA 500
Voer     TACATCTACAAAGCCTGCCAACACCATATCTACATGTTTCAGACGGTGGCAAGTACTTACAAACGTACGGAATGTGGATTCTTCAAACTCCAGATAAAAA 500
S288C    TACATCTACAAAGCCTGCCAACACCATATCTACATGTTTCAGACGGTGGCAAGTACTTACAAACGTACGGAATGTGGATTCTTCAAACTCCAGATAAAAA 500

         ★       510       520       530       540       550       560       570       580       590       600
WS34-70  AATGGACTAATTGGTCAATTGCTAGAGGTATGGTTGTAGATGACAAGCATATCACTGGTCTGGTAATTAAACCACAACATATTAGACAAATTGCTGACTC 600
Odin     AATGGACTAATTGGTCAATTGCTAGAGGTATGGTTGTAGATGACAAGCATATCACTGGTCTGGTAATTAAACCACAACATATTAGACAAATTGCTGACTC 600
Thor     A-TGGACTAATTGGTCAATTGCTAGAGGTATGGTTGTAGATGACAAGCATATCACTGGTCTGGTAATTAAACCACAACATATTAGACAAATTGCTGACTC 599
Freya    A-TGGACTAATTGGTCAATTGCTAGAGGTATGGTTGTAGATGACAAGCATATCACTGGTCTGGTAATTAAACCACAACATATTAGACAAATTGCTGACTC 599
Voer     A-TGGACTAATTGGTCAATTGCTAGAGGTATGGTTGTAGATGACAAGCATATCACTGGTCTGGTAATTAAACCACAACATATTAGACAAATTGCTGACTC 599
S288C    A-TGGACTAATTGGTCAATTGCTAGAGGTATGGTTGTAGATGACAAGCATATCACTGGTCTGGTAATTAAACCACAACATATTAGACAAATTGCTGACTC 599

                 610       620       630       640       650       660       670       680       690       700
WS34-70  TTGGGCAGCAATTGGAAAAGCAAATGAAATTCCTTTCGCGTTATGTTTTGGCGTTCCCCCAGCAGCTATTTTAGTTAGTTCCATGCCAATTCCTGAAGGT 700
Odin     TTGGGCAGCAATTGGAAAAGCAAATGAAATTCCTTTCGCGTTATGTTTTGGCGTTCCCCCAGCAGCTATTTTAGTTAGTTCCATGCCAATTCCTGAAGGT 700
Thor     TTGGGCAGCAATTGGAAAAGCAAATGAAATTCCTTTCGCGTTATGTTTTGGCGTTCCCCCAGCAGCTATTTTAGTTAGTTCCATGCCAATTCCTGAAGGT 699
Freya    TTGGGCAGCAATTGGAAAAGCAAATGAAATTCCTTTCGCGTTATGTTTTGGCGTTCCCCCAGCAGCTATTTTAGTTAGTTCCATGCCAATTCCTGAAGGT 699
Voer     TTGGGCAGCAATTGGAAAAGCAAATGAAATTCCTTTCGCGTTATGTTTTGGCGTTCCCCCAGCAGCTATTTTAGTTAGTTCCATGCCAATTCCTGAAGGT 699
S288C    TTGGGCAGCAATTGGAAAAGCAAATGAAATTCCTTTCGCGTTATGTTTTGGCGTTCCCCCAGCAGCTATTTTAGTTAGTTCCATGCCAATTCCTGAAGGT 699

                 710       720       730       740       750       760       770       780       790       800
WS34-70  GTTTCTGAATCGGATTATGTTGGCGCAATCTTGGGTGAGTCGGTTCCAGTAGTAAAATGTGAGACCAACGATTTAATGGTTCCTGCAACGAGTGAGATGG 800
Odin     GTTTCTGAATCGGATTATGTTGGCGCAATCTTGGGTGAGTCGGTTCCAGTAGTAAAATGTGAGACCAACGATTTAATGGTTCCTGCAACGAGTGAGATGG 800
Thor     GTTTCTGAATCGGATTATGTTGGCGCAATCTTGGGTGAGTCGGTTCCAGTAGTAAAATGTGAGACCAACGATTTAATGGTTCCTGCAACGAGTGAGATGG 799
Freya    GTTTCTGAATCGGATTATGTTGGCGCAATCTTGGGTGAGTCGGTTCCAGTAGTAAAATGTGAGACCAACGATTTAATGGTTCCTGCAACGAGTGAGATGG 799
Voer     GTTTCTGAATCGGATTATGTTGGCGCAATCTTGGGTGAGTCGGTTCCAGTAGTAAAATGTGAGACCAACGATTTAATGGTTCCTGCAACGAGTGAGATGG 799
S288C    GTTTCTGAATCGGATTATGTTGGCGCAATCTTGGGTGAGTCGGTTCCAGTAGTAAAATGTGAGACCAACGATTTAATGGTTCCTGCAACGAGTGAGATGG 799

                 810       820       830       840       850       860       870       880       890       900
WS34-70  TATTTGAGGGTACTTTGTCCTTAACAGATACACATCTGGAAGGCCCATTTGGTGAGATGCATGGATATGTTTTCAAAAGCCAAGGTCATCCTTGTCCATT 900
Odin     TATTTGAGGGTACTTTGTCCTTAACAGATACACATCTGGAAGGCCCATTTGGTGAGATGCATGGATATGTTTTCAAAAGCCAAGGTCATCCTTGTCCATT 900
Thor     TATTTGAGGGTACTTTGTCCTTAACAGATACACATCTGGAAGGCCCATTTGGTGAGATGCATGGATATGTTTTCAAAAGCCAAGGTCATCCTTGTCCATT 899
Freya    TATTTGAGGGTACTTTGTCCTTAACAGATACACATCTGGAAGGCCCATTTGGTGAGATGCATGGATATGTTTTCAAAAGCCAAGGTCATCCTTGTCCATT 899
Voer     TATTTGAGGGTACTTTGTCCTTAACAGATACACATCTGGAAGGCCCATTTGGTGAGATGCATGGATATGTTTTCAAAAGCCAAGGTCATCCTTGTCCATT 899
S288C    TATTTGAGGGTACTTTGTCCTTAACAGATACACATCTGGAAGGCCCATTTGGTGAGATGCATGGATATGTTTTCAAAAGCCAAGGTCATCCTTGTCCATT 899
```

```
                910       920       930       940       950       960       970       980       990      1000
WS34-70   GTACACTGTCAAGGCTATGAGTTACAGAGACAATGCTATTCTACCTGTTTCGAACCCCGGTCTTTGTACGGATGAGACACATACCTTGATTGGTTCACTA 1000
Odin      GTACACTGTCAAGGCTATGAGTTACAGAGACAATGCTATTCTACCTGTTTCGAACCCCGGTCTTTGTACGGATGAGACACATACCTTGATTGGTTCACTA 1000
Thor      GTACACTGTCAAGGCTATGAGTTACAGAGACAATGCTATTCTACCTGTTTCGAACCCCGGTCTTTGTACGGATGAGACACATACCTTGATTGGTTCACTA 999
Freya     GTACACTGTCAAGGCTATGAGTTACAGAGACAATGCTATTCTACCTGTTTCGAACCCCGGTCTTTGTACGGATGAGACACATACCTTGATTGGTTCACTA 999
Voer      GTACACTGTCAAGGCTATGAGTTACAGAGACAATGCTATTCTACCTGTTTCGAACCCCGGTCTTTGTACGGATGAGACACATACCTTGATTGGTTCACTA 999
S288C     GTACACTGTCAAGGCTATGAGTTACAGAGACAATGCTATTCTACCTGTTTCGAACCCCGGTCTTTGTACGGATGAGACACATACCTTGATTGGTTCACTA 999

               1010      1020      1030      1040      1050      1060      1070      1080      1090      1100
WS34-70   GTGGCTACTGAGGCCAAGGAGCTGGCTATTGAATCTGGCTTGCCAATTCTGGATGCCTTTATGCCTTATGAGGCTCAGGCTCTTTGGCTTATCTTAAAGG 1100
Odin      GTGGCTACTGAGGCCAAGGAGCTGGCTATTGAATCTGGCTTGCCAATTCTGGATGCCTTTATGCCTTATGAGGCTCAGGCTCTTTGGCTTATCTTAAAGG 1100
Thor      GTGGCTACTGAGGCCAAGGAGCTGGCTATTGAATCTGGCTTGCCAATTCTGGATGCCTTTATGCCTTATGAGGCTCAGGCTCTTTGGCTTATCTTAAAGG 1099
Freya     GTGGCTACTGAGGCCAAGGAGCTGGCTATTGAATCTGGCTTGCCAATTCTGGATGCCTTTATGCCTTATGAGGCTCAGGCTCTTTGGCTTATCTTAAAGG 1099
Voer      GTGGCTACTGAGGCCAAGGAGCTGGCTATTGAATCTGGCTTGCCAATTCTGGATGCCTTTATGCCTTATGAGGCTCAGGCTCTTTGGCTTATCTTAAAGG 1099
S288C     GTGGCTACTGAGGCCAAGGAGCTGGCTATTGAATCTGGCTTGCCAATTCTGGATGCCTTTATGCCTTATGAGGCTCAGGCTCTTTGGCTTATCTTAAAGG 1099

               1110      1120      1130      1140      1150      1160      1170      1180      1190      1200
WS34-70   TGGATTTGAAAGGGCTGCAAGCATTGAAGACAACGCCTGAAGAATTTTGTAAGAAGGTAGGTGATATTTACTTTAGGACAAAAGTTGGTTTTATAGTCCA 1200
Odin      TGGATTTGAAAGGGCTGCAAGCATTGAAGACAACGCCTGAAGAATTTTGTAAGAAGGTAGGTGATATTTACTTTAGGACAAAAGTTGGCTTTATAGTCCA 1200
Thor      TGGATTTGAAAGGGCTGCAAGCATTGAAGACAACGCCTGAAGAATTTTGTAAGAAGGTAGGTGATATTTACTTTAGGACAAAAGTTGGTTTTATAGTCCA 1199
Freya     TGGATTTGAAAGGGCTGCAAGCATTGAAGACAACGCCTGAAGAATTTTGTAAGAAGGTAGGTGATATTTACTTTAGGACAAAAGTTGGTTTTATAGTCCA 1199
Voer      TGGATTTGAAAGGGCTGCAAGCATTGAAGACAACGCCTGAAGAATTTTGTAAGAAGGTAGGTGATATTTACTTTAGGACAAAAGTTGGTTTTATAGTCCA 1199
S288C     TGGATTTGAAAGGGCTGCAAGCATTGAAGACAACGCCTGAAGAATTTTGTAAGAAGGTAGGTGATATTTACTTTAGGACAAAAGTTGGTTTTATAGTCCA 1199

               1210      1220      1230      1240      1250      1260      1270      1280      1290      1300
WS34-70   TGAAATAATTTTGGTGGCAGATGATATCGACATATTTAACTTCAAAGAAGTCATCTGGGCCTACGTTACAAGACATACACCTGTTGCAGATCAGATGGCT 1300
Odin      TGAAATAATTTTGGTGGCAGATGATATCGACATATTTAACTTCAAAGAAGTCATCTGGGCCTACGTTACAAGACATACACCTGTTGCAGATCAGATGGCT 1300
Thor      TGAAATAATTTTGGTGGCAGATGATATCGACATATTTAACTTCAAAGAAGTCATCTGGGCCTACGTTACAAGACATACACCTGTTGCAGATCAGATGGCT 1299
Freya     TGAAATAATTTTGGTGGCAGATGATATCGACATATTTAACTTCAAAGAAGTCATCTGGGCCTACGTTACAAGACATACACCTGTTGCAGATCAGATGGCT 1299
Voer      TGAAATAATTTTGGTGGCAGATGATATCGACATATTTAACTTCAAAGAAGTCATCTGGGCCTACGTTACAAGACATACACCTGTTGCAGATCAGATGGCT 1299
S288C     TGAAATAATTTTGGTGGCAGATGATATCGACATATTTAACTTCAAAGAAGTCATCTGGGCCTACGTTACAAGACATACACCTGTTGCAGATCAGATGGCT 1299

               1310      1320      1330      1340      1350      1360      1370      1380      1390      1400
WS34-70   TTTGATGATGTCACTTCTTTTCCTTTGGCTCCCTTTGTTTCGCAGTCATCCAGAAGTAAGACTATGAAAGGTGGAAAGTGCGTTACTAATTGCATATTTA 1400
Odin      TTTGATGATGTCACTTCTTTTCCTTTGGCTCCCTTTGTTTCGCAGTCATCCAGAAGTAAGACTATGAAAGGTGGAAAGTGCGTTACTAACTGCATATTTA 1400
Thor      TTTGATGATGTCACTTCTTTTCCTTTGGCTCCCTTTGTTTCGCAGTCATCCAGAAGTAAGACTATGAAAGGTGGAAAGTGCGTTACTAATTGCATATTTA 1399
Freya     TTTGATGATGTCACTTCTTTTCCTTTGGCTCCCTTTGTTTCGCAGTCATCCAGAAGTAAGACTATGAAAGGTGGAAAGTGCGTTACTAATTGCATATTTA 1399
Voer      TTTGATGATGTCACTTCTTTTCCTTTGGCTCCCTTTGTTTCGCAGTCATCCAGAAGTAAGACTATGAAAGGTGGAAAGTGCGTTACTAATTGCATATTTA 1399
S288C     TTTGATGATGTCACTTCTTTTCCTTTGGCTCCCTTTGTTTCGCAGTCATCCAGAAGTAAGACTATGAAAGGTGGAAAGTGCGTTACTAATTGCATATTTA 1399

               1410      1420      1430      1440      1450      1460      1470      1480      1490      1500
WS34-70   GACAGCAATACGAGCGCAGTTTTGACTACATAACTTGTAATTTTGAAAAGGGATATCCAAAAGGATTAGTTGACAAAGTAAATGAAAATTGGAAAAGGTA 1500
Odin      GACAGCAATATGAGCGCAGTTTTGACTACATAACTTGTAATTTTGAAAAGGGATATCCAAAAGGATTAGTTGACAAAGTAAATGAAAATTGGAAAAGGTA 1500
Thor      GACAGCAATATGAGCGCAGTTTTGACTACATAACTTGTAATTTTGAAAAGGGATATCCAAAAGGATTAGTTGACAAAGTAAATGAAAATTGGAAAAGGTA 1499
Freya     GACAGCAATATGAGCGCAGTTTTGACTACATAACTTGTAATTTTGAAAAGGGATATCCAAAAGGATTAGTTGACAAAGTAAATGAAAATTGGAAAAGGTA 1499
Voer      GACAGCAATATGAGCGCAGTTTTGACTACATAACTTGTAATTTTGAAAAGGGATATCCAAAAGGATTAGTTGACAAAGTAAATGAAAATTGGAAAAGGTA 1499
S288C     GACAGCAATATGAGCGCAGTTTTGACTACATAACTTGTAATTTTGAAAAGGGATATCCAAAAGGATTAGTTGACAAAGTAAATGAAAATTGGAAAAGGTA 1499

               1510
WS34-70   CGGATATAAATAA                                                                                        1513
Odin      CGGATATAAATAA                                                                                        1513
Thor      CGGATATAAATAA                                                                                        1512
Freya     CGGATATAAATAA                                                                                        1512
Voer      CGGATATAAATAA                                                                                        1512
S288C     CGGATATAAATAA                                                                                        1512
```

Figure S4 Alignment of *FDC1* ORF sequences. Sequences of the *FDC1* ORFs of Weihenstephan W34/70, Odin, Thor, Freya, Vör and S288C were aligned with DNASTAR MegAlign V12.1.0 (DNASTAR, Madison, WI, USA). Residues matching to *Freya* are shaded in black. The insertion of an A residue at position 502 in WS34/70 and Odin, which introduces a frameshift is marked by an asterisk. Accession numbers of HO sequences: WS34/70: AZAA01000000, Odin: PQ154477, Thor: PQ154478, Freya: PQ154479, Vör: PQ154480, S288C: NM_001180847.

```
                 10        20        30        40        50        60        70        80        90       100       110
WS34/70  MRKLNPALEFRDFIQVLKDEDDLIEITEEIDPNLEVGAIMRKAYESHLPAPLFKNLKGASKDLFSILGCPAGLRSKEKGDHGRIAHHLGLDPKTTIKEIIDYLLECKEKE 110
Odin     MRKLNPALEFRDFIQVLKDEDDLIEITEEIDPNLEVGAIMRKAYESHLPAPLFKNLKGASKDLFSILGCPAGLRSKEKGDHGRIAHHLGLDPKTTIKEIIDYLLECKEKE 110
Freya    MRKLNPALEFRDFIQVLKDEDDLIEITEEIDPNLEVGAIMRKAYESHLPAPLFKNLKGASKDLFSILGCPAGLRSKEKGDHGRIAHHLGLDPKTTIKEIIDYLLECKEKE 110
Thor     MRKLNPALEFRDFIQVLKDEDDLIEITEEIDPNLEVGAIMRKAYESHLPAPLFKNLKGASKDLFSILGCPAGLRSKEKGDHGRIAHHLGLDPKTTIKEIIDYLLECKEKE 110
Vör      MRKLNPALEFRDFIQVLKDEDDLIEITEEIDPNLEVGAIMRKAYESHLPAPLFKNLKGASKDLFSILGCPAGLRSKEKGDHGRIAHHLGLDPKTTIKEIIDYLLECKEKE 110
S288C    MRKLNPALEFRDFIQVLKDEDDLIEITEEIDPNLEVGAIMRKAYESHLPAPLFKNLKGASKDLFSILGCPAGLRSKEKGDHGRIAHHLGLDPKTTIKEIIDYLLECKEKE 110

                120       130       140       150       160       170       180       190       200       210       220
WS34/70  PLPPITVPVSSAPCKTHILSEEKIHLQSLPTPYLYVSDGGKYLQTYGMWILQTPDKKMD.                                                   170
Odin     PLPPITVPVSSAPCKTHILSEEKIHLQSLPTPYLHVSDGGKYLQTYGMWILQTPDKKMD.                                                   170
Freya    PLPPITVPVSSAPCKTHILSEEKIHLQSLPTPYLHVSDGGKYLQTYGMWILQTPDKKWTNWSIARGMVVDDKHITGLVIKPQHIRQIADSWAAIGKANEIPFALCFGVPP 220
Thor     PLPPITVPVSSAPCKTHILSEEKIHLQSLPTPYLHVSDGGKYLQTYGMWILQTPDKKWTNWSIARGMVVDDKHITGLVIKPQHIRQIADSWAAIGKANEIPFALCFGVPP 220
Vör      PLPPITVPVSSAPCKTHILSEEKIHLQSLPTPYLHVSDGGKYLQTYGMWILQTPDKKWTNWSIARGMVVDDKHITGLVIKPQHIRQIADSWAAIGKANEIPFALCFGVPP 220
S288C    PLPPITVPVSSAPCKTHILSEEKIHLQSLPTPYLHVSDGGKYLQTYGMWILQTPDKKWTNWSIARGMVVDDKHITGLVIKPQHIRQIADSWAAIGKANEIPFALCFGVPP 220

                230       240       250       260       270       280       290       300       310       320       330
WS34/70                                                                                                                 170
Odin                                                                                                                    170
Freya    AAILVSSMPIPEGVSESDYVGAILGESVPVVKCETNDLMVPATSEMVFEGTLSLTDTHLEGPFGEMHGYVFKSQGHPCPLYTVKAMSYRDNAILPVSNPGLCTDETHTLI 330
Thor     AAILVSSMPIPEGVSESDYVGAILGESVPVVKCETNDLMVPATSEMVFEGTLSLTDTHLEGPFGEMHGYVFKSQGHPCPLYTVKAMSYRDNAILPVSNPGLCTDETHTLI 330
Vör      AAILVSSMPIPEGVSESDYVGAILGESVPVVKCETNDLMVPATSEMVFEGTLSLTDTHLEGPFGEMHGYVFKSQGHPCPLYTVKAMSYRDNAILPVSNPGLCTDETHTLI 330
S288C    AAILVSSMPIPEGVSESDYVGAILGESVPVVKCETNDLMVPATSEMVFEGTLSLTDTHLEGPFGEMHGYVFKSQGHPCPLYTVKAMSYRDNAILPVSNPGLCTDETHTLI 330

                340       350       360       370       380       390       400       410       420       430       440
WS34/70                                                                                                                 170
Odin                                                                                                                    170
Freya    GSLVATEAKELAIESGLPILDAFMPYEAQALWLILKVDLKGLQALKTTPEEFCKKVGDIYFRTKVGFIVHEIILVADDIDIFNFKEVIWAYVTRHTPVADQMAFDDVTSF 440
Thor     GSLVATEAKELAIESGLPILDAFMPYEAQALWLILKVDLKGLQALKTTPEEFCKKVGDIYFRTKVGFIVHEIILVADDIDIFNFKEVIWAYVTRHTPVADQMAFDDVTSF 440
Vör      GSLVATEAKELAIESGLPILDAFMPYEAQALWLILKVDLKGLQALKTTPEEFCKKVGDIYFRTKVGFIVHEIILVADDIDIFNFKEVIWAYVTRHTPVADQMAFDDVTSF 440
S288C    GSLVATEAKELAIESGLPILDAFMPYEAQALWLILKVDLKGLQALKTTPEEFCKKVGDIYFRTKVGFIVHEIILVADDIDIFNFKEVIWAYVTRHTPVADQMAFDDVTSF 440

                450       460       470       480       490       500
WS34/70                                                                                                                 170
Odin                                                                                                                    170
Freya    PLAPFVSQSSRSKTMKGGKCVTNCIFRQQYERSFDYITCNFEKGYPKGLVDVKVNENWKRYGYK.                                              504
Thor     PLAPFVSQSSRSKTMKGGKCVTNCIFRQQYERSFDYITCNFEKGYPKGLVDVKVNENWKRYGYK.                                              504
Vör      PLAPFVSQSSRSKTMKGGKCVTNCIFRQQYERSFDYITCNFEKGYPKGLVDVKVNENWKRYGYK.                                              504
S288C    PLAPFVSQSSRSKTMKGGKCVTNCIFRQQYERSFDYITCNFEKGYPKGLVDVKVNENWKRYGYK.                                              504
```

Figure S5 Alignment of Fdc1 protein sequences. Fdc1 protein sequenced were translated from the ORF sequences shown in Figure S4. Residues matching to Freya are shaded in black. Note: the frameshift mutation in WS34/70 and Odin leads to a downstream stop codon and a shortened protein.
